# Elementary processes of DNA surface hybridization resolved by single-molecule kinetics: implication for macroscopic device performance†

**DOI:** 10.1039/d0sc04449k

**Published:** 2020-12-22

**Authors:** Takanori Harashima, Yusuke Hasegawa, Satoshi Kaneko, Yuki Jono, Shintaro Fujii, Manabu Kiguchi, Tomoaki Nishino

**Affiliations:** Department of Chemistry, School of Science, Tokyo Institute of Technology 2-12-1 W4-11 Ookayama Meguro-ku Tokyo 152-8551 Japan tnishino@chem.titech.ac.jp

## Abstract

Direct monitoring of single-molecule reactions has recently become a promising means of mechanistic investigation. However, the resolution of reaction pathways from single-molecule experiments remains elusive, primarily because of interference from extraneous processes such as bulk diffusion. Herein, we report a single-molecule kinetic investigation of DNA hybridization on a metal surface, as an example of a bimolecular association reaction. The tip of the scanning tunneling microscope (STM) was functionalized with single-stranded DNA (ssDNA), and hybridization with its complementary strand on an Au(111) surface was detected by the increase in the electrical conductance associated with the electron transport through the resulting DNA duplex. Kinetic analyses of the conductance changes successfully resolved the elementary processes, which involve not only the ssDNA strands and their duplex but also partially hybridized intermediate strands, and we found an increase in the hybridization efficiency with increasing the concentration of DNA in contrast to the knowledge obtained previously by conventional ensemble measurements. The rate constants derived from our single-molecule studies provide a rational explanation of these findings, such as the suppression of DNA melting on surfaces with higher DNA coverage. The present methodology, which relies on intermolecular conductance measurements, can be extended to a range of single-molecule reactions and to the exploration of novel chemical syntheses.

## Abstract 2

Hybridization of a single DNA molecule on a surface was investigated by electrical conductance measurements. The hybridization efficiency increases with increasing the DNA concentration, in contrast to preceding studies with ensemble studies.

## Graphical abstract

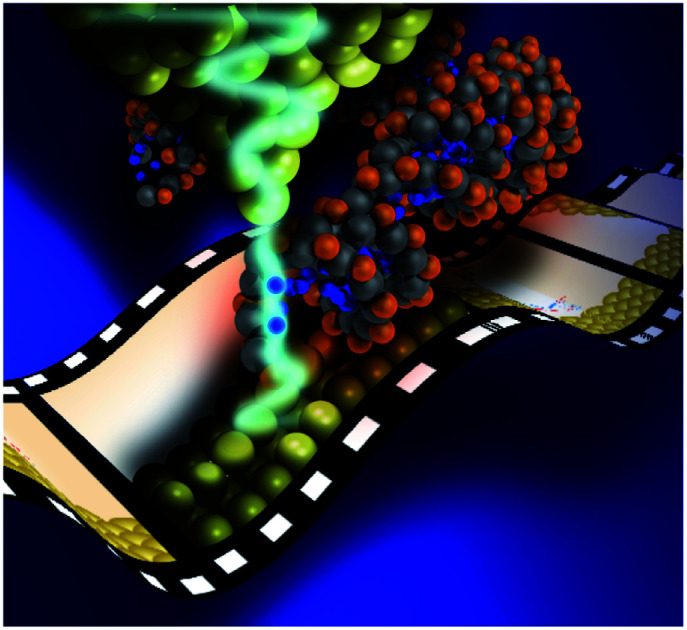

## 1. Introduction

There have been significant recent advances in the methodology of single-molecule investigation. Considerable effort has been devoted to exploring the electronic properties of single molecules by scanning tunneling microscopy (STM) and mechanically controllable break-junctions (BJ).^1–3^ The measurements of the single-molecule conductance have been conducted in pursuit of molecular electronics, which exploits single molecules as components for the electronic functions.^4^ From the viewpoint of fundamental research, electronic measurements have been also utilized in tracking the chemical processes of a single molecule.^5,6^ The chemical reaction can be detected based on the single-molecule conductance modulated by the chemical transformation of the target molecule. Biochemical processes of a single molecule, in particular, are thoroughly investigated using the conductance measurements as well as fluorescence-based assays, taking advantage of the ease of fluorescent labeling and the larger molecular dimensions.^7–9^ For example, the stepping motions of a single motor protein have been observed directly by total internal reflection fluorescence microscopy.^10,11^ Dissociation of single DNA duplexes has been studied by STM^12^ and atomic force microscopy.^13–16^ Single-molecule investigations have provided mechanistic insights into chemical reactions and biological processes hidden in conventional ensemble measurements.^17,18^ Despite significant advances in single-molecule reaction methodology, experimental determination of reaction pathways leading from initial interactions through reaction intermediates to final products has yet to be accomplished. Elucidation of reaction pathways and identification of elementary processes will lead to an unprecedented understanding of reaction mechanisms (*e.g.*, stochastic behavior, microscopic environmental effects) and will promote the discovery of novel chemical reactivity.

In this study, we investigate the hybridization of single DNA molecules as an archetypal example of a bimolecular association reaction. Hybridization was monitored *via* the tunneling current measured by STM. An STM tip was functionalized with single-stranded DNA (ssDNA) to induce hybridization with its complement adsorbed on Au(111) substrate. Increases in the electrical conductance were detected upon the onset of the hybridization of these strands. Kinetic analysis of time-dependent changes in conductance revealed elementary steps in the course of hybridization and dehybridization. Processes identified include the formation of a partially hybridized DNA intermediate that cannot be distinguished from the fully dissociated state based on conductance alone. We found that the hybridization efficiency estimated from single-molecule measurements is entirely different from that reported by ensemble measurements. Our results provide fruitful insights toward improving the performance of DNA-based devices and offer a unique blueprint for elucidating the reaction pathways of single-molecule chemical reactions.

## 2. Experimental

### 2.1 Sample preparation

Ultraflat gold films, prepared with thermally evaporated Au films (150 nm thick) on mica,^19^ were employed as Au(111) substrates. STM tips were prepared by mechanically cutting Au wires (99.999%, 0.25 mm diameter). The 90-nucleotide-long ssDNA having the sequence of 5′-GCG CAA TGA AAG AAG CCC GTG CCG TTA TCA GGC CGG ATT AGG TTA GAA TCG TGG AGC CAT TCC ACA TCC GCT TGT GGT TTG ACG GCC ACC-3′ and its complementary strand were used as samples. The former was modified with a –(CH_2_)_3_SH linker at the 3′ terminus and immobilized on the substrates. The latter was modified with the same linker at the 5′ terminus and was used to functionalize the STM tips. The diluted sample surface was prepared by immersing the substrate in 20 nM ssDNA solution for 10 min. A 1 μM ssDNA solution and immersion times of 10 min and 2 h were used to prepare the dense and ordered sample surfaces, respectively. The STM tip was functionalized by immersion in 1 μM ssDNA for 2 h. The prolonged immersion of the tip from 2 to 24 h afforded results identical to those obtained with the tip prepared by the aforementioned condition, indicating the nearly saturated coverage of ssDNA on the tip. The substrates and tips were carefully washed with copious amounts of water after immersion.

### 2.2 Tunneling current measurements

Current measurements were performed with an SPM 5100 system (Agilent Technologies, Santa Clara, CA) in air at room temperature. The sampling frequency was 20 kHz, and the applied bias voltage was 20 mV. The ssDNA molecular tip was approached to, but never contacted with, the sample surface, and a constant tunneling gap determined by the set-point current was established *via* the STM feedback loop. The STM scanner was allowed to settle for at least 4 h before data acquisition to suppress unwanted thermal drift. The current traces were obtained for 2 s with the feedback loop disabled. The measurements were repeated at 0.5 s intervals with feedback reactivated between each trace. More than one thousand traces were recorded in a single session.

## 3. Results

### 3.1 *In situ* single-molecule detection of DNA hybridization

**Fig. 1:**
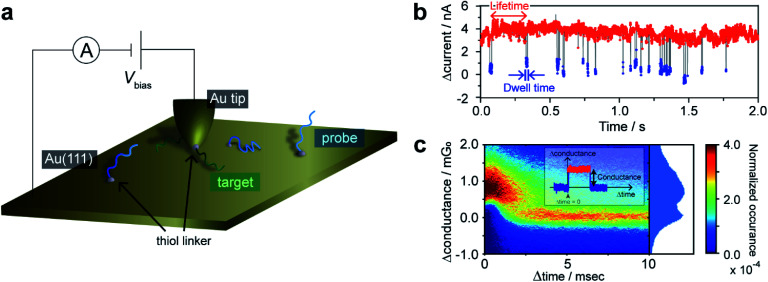
STM *I*–*t* measurements for single-molecule detection of hybridization. (a) Schematic illustration of the current measurements. (b) Typical time trace showing the high- and low-conducting states. Bias voltage, 20 mV; set-point current, 16 nA. (c) 2D histogram of plateaus detected in the current traces. The origin of the time (or conductance) axis is set at the onset of each plateau (or at the baseline current).

An essential aspect of kinetic investigations is the detection of chemical processes or reactions as a function of time. Herein, we utilized STM current–time (*I*–*t*) measurements^20,21^ to monitor DNA hybridization on a single-molecule basis. It is first necessary to demonstrate that hybridization of a single DNA molecule is accompanied by a detectable change in tunneling current. Au(111) substrate was modified with 90-nucleotide-long ssDNA having a thiol linker introduced at the 3′ terminus *via* Au–S chemisorption. An STM gold tip was modified with a complementary ssDNA strand *via* a thiol linker at the 5′ terminus (Fig. 1a). The introduction of the thiol linkers at different termini of the DNA strands on the tip and substrate allows us to employ the long DNA for the measurement of single-molecule conductance.^12^ In our previous work, we demonstrated that a single-molecule junction comprising long DNA exhibits self-restoring capabilities arising from the superior thermal stability of the long duplex even under the application of external mechanical perturbation.^12^ However, in the present kinetic study, the use of long DNA is vital given that very short duplexes, used in conventional studies on the single-molecule conductance, have been known to show kinetics different from that of longer, natural DNAs.^22,23^

The functionalized STM tip was brought close to the sample surface and held in a stationary position to record the tunneling current. The resulting current trace exhibits characteristic current plateaus consisting of steep increases and subsequent decreases in the tunneling current (Fig. 1b). The plateaus have been attributed to the formation of molecular junctions.^21^ The molecular bridging in the junction structure causes the current increase, while the junction breakdown results in the decrease in the current to the initial set-point value. The plateaus were detected by adaptive threshold analysis (ATA),^24^ which analyzes the *I*–*t* traces based on a recursive low-pass filtering function.^5^ A two-dimensional (2D) histogram shows that the statistically most probable plateau appears at 1.3 mG_0_ (Fig. 1c and ESI S1†). This conductance value agrees well with the single-molecule conductance of the double-stranded DNA (dsDNA) determined by STM-BJ measurements (ESI S2†). No plateau was observed for the Au surface without ssDNA modification, which precludes the possibility that the plateaus are due to non-specific adsorption of the ssDNA on the STM tip to the substrate. Furthermore, we performed control experiments where the tip and substrate were modified with non-complementary ssDNAs (ESI S3†). Again, essentially no plateau was detected, indicating that the mere modification of the tip and substrate surfaces with ssDNAs cannot account for these plateaus. We, therefore, conclude that the plateaus result from the formation of the single molecule of dsDNA produced by hybridization of the DNA strands on the STM tip and substrate. The sudden decrease in current at the end of plateaus is attributed to the breakdown of the DNA single-molecule junction. We have demonstrated in a previous study^12,25^ that the breakdown involves dehybridization of the DNA duplex and not desorption of the thiol from the STM tip or substrate. Furthermore, we have shown that presence of a single-base mismatch in dsDNA leads to an order of magnitude decrease in the conductance of the molecular junction.^26^ Thus, the current decrease at the plateau end could be caused by dissociation of even a single base pair within dsDNA. Molecular dynamics (MD) simulations indicate that the dsDNA in the junction undergoes partial dehybridization at the onset of the breakdown process (ESI S4†). We investigated the possibility that junction breakdown is caused by the external force exerted by the STM on the dsDNA in the junction by varying the gap width and set-point currents (ESI S4 and S5†). We found at large set-point currents, and thus small gap widths, that the effect of the mechanical force exerted by the STM tip is negligible and conclude that thermal activation is responsible for the junction breakdown. Therefore, the set-point currents were chosen to ensure the observation of spontaneous hybridization/dehybridization, from which the intrinsic kinetics of the process are determined. The tip–sample distance under this experimental condition was estimated to be 0.69 nm according to the reported procedure (ESI S6†).^27^ In the following sections, the single-molecule kinetics of DNA hybridization on the surface are discussed in terms of the lifetime and dwell time of the *I*–*t* traces (Fig. 1b). The lifetime and dwell time reflect the kinetics of dissociation and formation of the DNA duplex, respectively.

### 3.2 Dehybridization kinetics by lifetime analysis

**Fig. 2:**
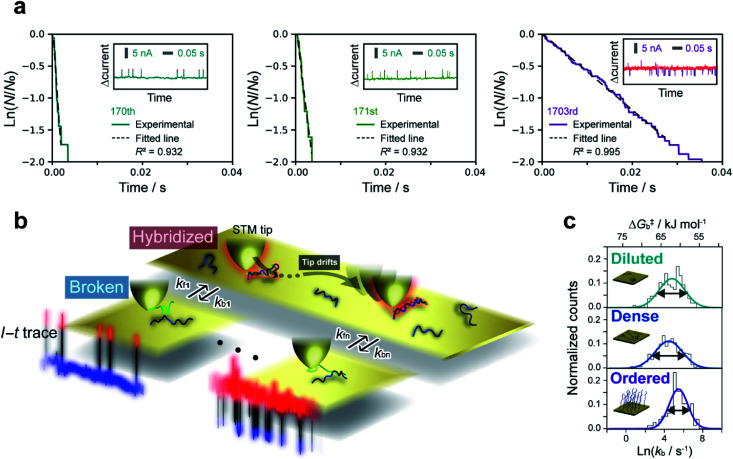
Kinetic lifetime analysis of *I*–*t* plateaus. (a) Reaction plots created from plateau lifetimes appearing in each current trace. Plots constructed from the 170th, 171st, and 1703rd traces among thousands of *I*–*t* measurements are shown as examples. Insets show the raw traces. *N* and *N*_0_ are the numbers of surviving and total plateaus, respectively. Bias voltage, 20 mV; set-point current, 16 nA. (b) Schematic illustration of fluctuations in the dehybridization rate constant. (c) Histograms of dehybridization rate constants for diluted (top), dense (middle), and ordered (bottom) sample surfaces. The histograms were fitted by a Gaussian function according to the Eyring–Polanyi equation.

The lifetime of each plateau in the *I*–*t* traces was extracted to assess the retention time of the dsDNA junction before its breakdown by dehybridization. To evaluate the dehybridization kinetics, the lifetime was used to construct a reaction plot showing the time course of the number of surviving plateaus (*N*) normalized by the total number of plateaus observed (*N*_0_). The resulting plots for each *I*–*t* trace are linear on a semilogarithmic scale (Fig. 2a) confirming the first-order kinetics of the dehybridization reaction. The dehybridization rate constant, *k*_b_, was determined from the slope of the plots. Despite the linearity of the plots for individual *I*–*t* traces, *k*_b_ gradually changes during the course of consecutive measurements (Fig. 2a). Autocorrelation analysis of *k*_b_ indicates a correlation decay of several seconds (ESI S7†). This interval is comparable to the time required for the STM tip to be displaced by a few atomic distances parallel to the substrate surface due to thermal drift of the STM instrument (ESI S8†). The variation of *k*_b_, therefore, may result from differences in the local environment around the dsDNA (see Fig. 2b).

To further assess the origin of the *k*_b_ variation, we prepared three different sample surfaces by immersing the Au substrate in different concentrations of ssDNA solution for different times. The coverage of the adsorbed ssDNA on each sample was estimated by electrochemical measurements (ESI S9†), and adsorbed structures were characterized by X-ray photoemission spectroscopy (ESI S10†). The structure of adsorbed ssDNA at the lowest and middle coverages (denoted as diluted and dense, respectively) is flat with the bases predominantly chemisorbed on the Au surface. In contrast, the almost fully covered (ordered) sample adopts a self-assembled upright structure.^28^ The characteristic plateaus shown in Fig. 1b were observed in the *I*–*t* traces measured for all three sample surfaces. The conductance of the plateaus was independent of the sample type and was consistent with the single-molecule conductance of dsDNA (ESI S1†), demonstrating that the single-molecule junctions of the dsDNA were detected in the current measurements for all the sample surfaces. The rate constants, *k*_b_, were then statistically evaluated on the three surface types (Fig. 2c). The mean value of *k*_b_ is nearly independent of the surface coverage and structure of adsorbed DNA, which is discussed further below in terms of the activation energy. Meanwhile, the full-width at half-maximum of the distribution of ln *k*_b_ is smallest for the ordered sample (3.1 ± 0.4, 3.4 ± 0.2, and 2.4 ± 0.2 for diluted, dense, and ordered surfaces, respectively). A similar result was obtained for the conductance of the DNA single-molecule junction (ESI S1†). We attribute the small dispersion in conductance and rate constant values of the ordered sample to the restricted configuration of the densely packed adlayer. The rate constant was used to evaluate the activation energy, Δ*G*^‡^, of the dehybridization reaction according to Eyring–Polanyi equation,
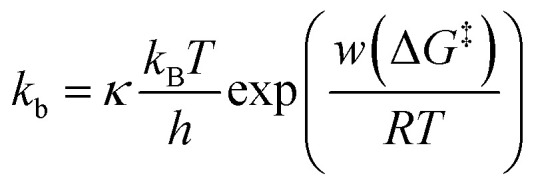
where *k*_B_, *h*, *R*, and *T* are the Boltzmann constant, Planck constant, gas constant, and temperature, respectively. We express Δ*G*^‡^ as a Gaussian function, *w*(Δ*G*^‡^), to reflect the variability of *k*_b_ (see above). Assuming a transmission coefficient, *κ*, of unity, as is frequently the case in single-molecule reactions,^20,29–31^ the average Δ*G*^‡^ for all sample surfaces is 61–62 kJ mol^−1^. The estimation based on Δ*G*^‡^ combined with MD simulations suggests that the dehybridization process is initiated from the dissociations of a few terminal base pairs (ESI S11†).

### 3.3 Kinetic modeling of hybridization by dwell-time analysis

**Fig. 3:**
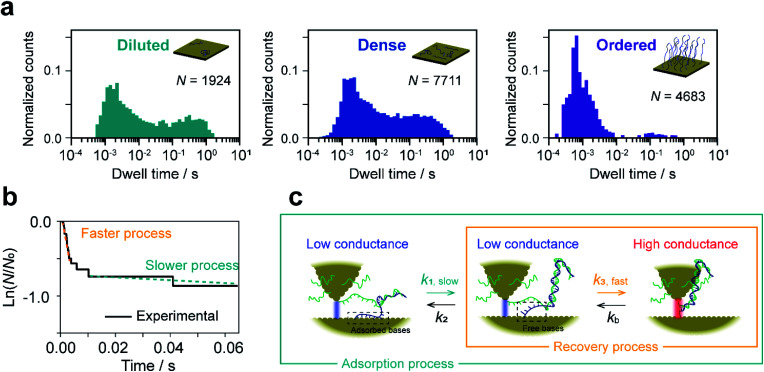
Kinetic analysis of dwell time between *I*–*t* plateaus. (a) Histograms of dwell time for diluted (left), dense (middle), and ordered (right) sample surfaces. (b) Reaction plots created from the dwell time of plateaus appearing in a single *I*–*t* trace. Bias voltage, 20 mV; set-point current, 16 nA. (c) Schematic drawing of the kinetic model constructed from the dwell-time analysis.

The dwell times of the *I*–*t* traces (Fig. 1b) were analyzed to characterize the hybridization process of single DNA molecules. Fig. 3a shows histograms of the dwell times for diluted, dense, and ordered sample surfaces. Two distributions are found in the 10^−3^ and 1 s range in all cases, although the longer component is significantly suppressed for the ordered surface. The reaction plots of dwell time (Fig. 3b) deviate from linearity unlike those in Fig. 2a. The non-linear plots preclude estimation of the hybridization rate constant, *k*_f_, and require kinetic modeling of the hybridization process to evaluate *k*_f_. We first consider the elementary processes comprising the kinetic model. The dwell-time histogram shows that the relative occurrence of the longer component with respect to the shorter one is nearly the same for the diluted and dense surfaces (Fig. 3a, left and middle, respectively), whereas the shorter component is more prominent for the ordered surface (Fig. 3a, right). Given the distribution of ssDNA surface conformations in the three samples, this observation suggests that the slower process involves ssDNA strands lying flat on the Au surface (the adsorption process in Fig. 3c). During the long dwell time, the dsDNA in the molecular junction fully dissociates and other pairs of ssDNA strands may hybridize to form the junction, which results in the plateau in the *I*–*t* trace. The estimation of Δ*G*^‡^ and the MD simulation (ESI S4†) invoked in assigning the short-lived component in Fig. 3a both suggest the temporary presence of a partially melted, incomplete duplex. Thus, we attribute the shorter component to the regeneration of the complete duplex from partially melted strands (the recovery process in Fig. 3c). To support this explanation, we evaluated the relationship between the dwell time and cross-correlation of conductance values of adjacent plateaus (ESI S12†). A short dwell time results in a high cross-correlation, whereas cross-correlation is nearly lost at long dwell times. This analysis coincides with the conclusion that the recovery process with a short dwell time regenerates the same DNA junction, which exhibits nearly the same conductance. Conversely, complete dissociation of the DNA duplex requires that other strands initially adsorbed on the substrate must hybridize to form the junction after a long dwell time (the adsorption process). The dsDNA in the two junctions before and after the dwell time may have different conformations and/or be in different local environments, leading to a change in single-molecule conductance and loss of cross-correlation. The kinetic model of the hybridization process is based on the elementary processes described above, which involves fully dissociated and partially melted states. The linear and non-linear appearances of the reaction plots for dehybridization and hybridization, which correspond to the lifetime and dwell time, respectively (Fig. 1b), lead to the sequential reaction scheme depicted in Fig. 3c (ESI S13†). In this model, fully dissociated ssDNAs on the STM tip and the substrate hybridize *via* partially hybridized states. Alternatively, dsDNA is re-established after partial dehybridization.

### 3.4 Estimation of elementary reaction rate constants

**Fig. 4:**
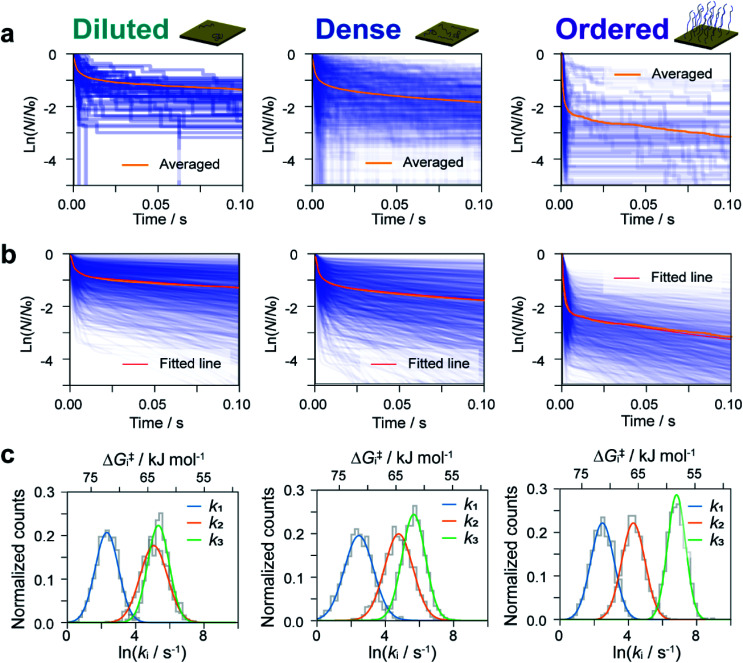
Simulation-based analyses of reaction plots. Experimental (a) and simulated (b) reaction plots of the dwell time for diluted (left), dense (middle), and ordered (right) sample surfaces. In (a), 56, 456, and 104 reaction plots are overlaid for diluted, dense, and ordered surfaces, respectively. One thousand simulated reaction plots are shown in (b). Orange and red lines show the averaged experimental and simulated reaction plots, respectively. (c) Distributions of *k*_1_, *k*_2_, and *k*_3_ determined from the fitting procedure.

We now estimate rate constants for each elementary step in the kinetic model established above. These steps include the sequential adsorption process (rate constants *k*_1_ and *k*_2_ for the forward and backward reactions, respectively; Fig. 3c) and the recovery process (*k*_3_ and *k*_b_ for the forward and backward reactions, respectively; Fig. 3c). Analogy with the *k*_b_ analysis in Fig. 2c suggests that rate constants *k*_1_, *k*_2_, and *k*_3_ will also follow a log-normal distribution reflecting fluctuations in the local environment of ssDNA arising from differences in the local surface concentration and interactions with the substrate surface. To obtain an accurate analysis, we carried out a simulation-based parameter estimation taking the distribution of rate constants into account. Fig. 4a shows reaction plots constructed from the experimentally measured dwell times. Overlaid plots of individual *I*–*t* traces and averaged plots of all traces are shown. Using Gaussian distributions of ln *k*_1_, ln *k*_2_, and ln *k*_3_ as fitting parameters, one thousand reaction plots were generated by simulation, and their average response was calculated for comparison with experimental results (see ESI S14† for detailed arguments). The simulated individual and averaged plots based on the distributions of ln *k*_1_, ln *k*_2_, and ln *k*_3_ are illustrated in Fig. 4b. Comparison of Fig. 4a and b clearly show an agreement between the simulated and experimental results, which verifies the estimated distribution of rate constants (Fig. 4c and S19†).

## 4. Discussion

**Fig. 5:**
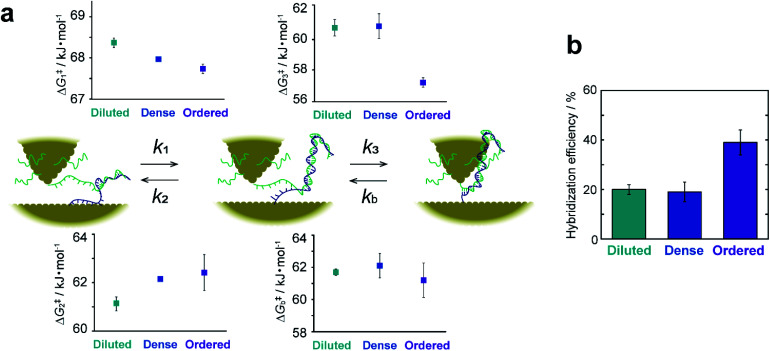
Kinetic description of elementary processes in hybridization. (a) Schematic illustration of elementary steps and their activation energies for diluted, dense, and ordered sample surfaces. (b) Bar graph of hybridization efficiencies calculated from the lifetime of *I*–*t* plateaus. Error bars show standard deviations derived from measurements on replicate substrates.

The rate constant and activation energy of each step in DNA hybridization have been successfully determined on a single-molecule basis by kinetic analysis of *I*–*t* measurements (Fig. 5a and ESI S15†). Previous investigations of hybridization kinetics have relied largely on fluorescence measurements.^32–38^ Hybridization efficiency, which is a direct measure of the hybridization equilibrium, is a principal difference between the present and earlier studies. Fig. 5b displays the hybridization efficiency determined as the total lifetime of dsDNA normalized by the entire duration of the *I*–*t* measurements. The efficiency of ordered samples with high DNA surface coverage is approximately twice that of samples with lower surface coverages. This observation contradicts the findings of previous studies, wherein efficiency increases with decreasing surface coverage of ssDNA.^32–38^ This inverse correlation has been attributed to the impeded diffusion of complementary ssDNA from bulk solution by electrostatic repulsion. These behaviors of the surface hybridization have been thoroughly investigated using thiolated DNAs using a range of electrochemical techniques.^33,36,39–42^ For example, the hybridization behaviors were analyzed under controlled electrostatic interactions between immobilized DNA and chemical species in solution and between immobilized DNAs. The results are summarized in a diagram of hybridization regimes showing the hybridization behavior for a particular surface coverage of DNA.^33^ Electrochemical studies using DNA with bulky hairpin structures have illustrated that steric hindrance of the adsorbed DNA also hinders the hybridization process with target DNA in the solution phase.^39,40^ These studies eventually led to the development of signal amplification strategies using DNA-functionalized nanoparticles.^41,42^ On the other hand, bulk diffusion is absent in the present method, because the ssDNA remains close to the surface where its complement has been modified (see Fig. 1a). We thus ascribe the seemingly contradictory results to the absence of diffusion from the bulk solution to the surface. The kinetic analyses provide rational explanation as discussed below. Besides, the difference illustrated in Fig. 5b demonstrates a specific advantage of the present work. This methodology facilitates the direct probing of interactions and chemical reactions involving surface-bound molecules without interference from bulk solution and enables the extraction of surface reaction kinetics on a single-molecule basis.

The favorable hybridization found at high surface coverage is explained by the activation energies, Δ*G*^‡^_*i*_ (*i* = 1–3 or *b*), calculated from the rate constants, *k*_*i*_, of the elementary processes. Fig. 5a shows that the activation energies of the forward (or backward) reactions are smaller (or larger) for the ordered sample than those for sample surfaces with a lower DNA coverage. This makes hybridization favorable. The partial dissociation of complete dsDNA is an exception. In this case, Δ*G*^‡^_*b*_ is nearly constant regardless of surface coverage, which indicates the insensitivity of the process to the local environment. The activation energies Δ*G*^‡^_1_ and Δ*G*^‡^_2_ associated with the formation and dissociation of partially hybridized strands decrease and increase, respectively, with increasing surface coverage. The decrease in Δ*G*^‡^_1_ reflects the increased probability that ssDNA collides with its complement at higher coverages. The increase in Δ*G*^‡^_2_ with increasing coverage is due to the stochastic blocking of vacant adsorption sites on the gold surface. Δ*G*^‡^_3_ decreases only for the ordered sample and is nearly equal for the diluted and dense surfaces. This difference in behavior suggests that the adlayer structure in terms of a flat or upright configuration (see ESI S10†), rather than coverage, controls hybridization. We hypothesized that a decrease in the interaction between the DNA and the substrate surface associated with the upright configuration causes the Δ*G*^‡^_3_ decrease. To test this assumption, the dense sample surface was further modified with 3-mercaptopropionic acid (MPA) to form a binary ssDNA/MPA adlayer. As shown in previous studies, the binary SAMs reduce the DNA–Au surface interaction and prevent nonspecific adsorption of ssDNA.^43–46^ The *I*–*t* measurements were performed, and their kinetic analyses revealed that Δ*G*^‡^_3_ indeed decreased on the ssDNA/MPA adlayer (ESI S16†). The results confirm that the surface interaction of ssDNA with the substrate surface is closely associated with Δ*G*^‡^_3_.

To summarize the kinetic analyses, the formation of the partially hybridized structure becomes favorable with increasing DNA surface coverage owing to the effect of Δ*G*^‡^_1_ and Δ*G*^‡^_2_, and the complete hybridization is facilitated for DNA strands with the upright configuration with high surface coverage owing to the effect of Δ*G*^‡^_3_. As a consequence, the surface hybridization proceeds efficiently upon increasing the surface coverage of DNA.

## 5. Conclusions

In summary, we have measured the hybridization reaction resulting in the formation of a single DNA duplex based on the time dependence of tunneling currents. Kinetic analysis reveals the elementary processes and enables a quantitative determination of rate constants and free energies. Our single-molecule kinetic study provides information regarding hybridization efficiency that differs from that estimated by ensemble measurements and reaction acceleration/deceleration phenomena under the influence of microscopic environments. These new insights should contribute to improved performance for many DNA-based devices. The methodology we have described can be extended to the investigation of intermolecular chemical reactions between a variety of single molecules and can lead to mechanistic understanding of chemical reactions and exploration of novel reactivity from a single-molecule perspective.

## Conflicts of interest

There are no conflicts to declare.

## Supplementary Material

SC-012-D0SC04449K-s001
