# Supplementary material for: Elementary processes of DNA surface hybridization resolved by single-molecule kinetics: implication for macroscopic device performance
Source: Chem Sci. 2020 Dec 22;12(6):2217–24. doi: 10.1039/d0sc04449k (PMC8179252; doi:10.1039/d0sc04449k)
Supplement: SC-012-D0SC04449K-s001 [file SC-012-D0SC04449K-s001.pdf]

Electronic Supplementary Material (ESI) for Chemical Science.
This journal is © The Royal Society of Chemistry 2020

Supplementary Information

Elementary Processes of DNA Surface Hybridization Resolved by Single-molecule Kinetics: Implication for Macroscopic Device Performance

Takanori Harashima, Yusuke Hasegawa, Satoshi Kaneko, Yuki Jono, Shintaro Fujii, Manabu Kiguchi and Tomoaki Nishino

Contents.

S1: Statistical analysis of plateaus in *I–t* traces. 3

S2: STM *G–z* measurements for single-molecule junction of dsDNA. 5

S3: *I–t* measurements using non-complementary DNAs. 8

S4: Molecular dynamics study of DNA melting under mechanical forces. 9

S5: Effect of mechanical force of tip on dehybridization kinetics. 14

S6: Estimation of the tip–sample distance in the *I–t* measurement. 17

S7: Memory effect of rate constant in consecutive *I–t* measurement. 19

S8: Evaluation of STM tip drift. 22

S9: Electrochemical measurements of the DNA-modified gold surfaces. 24

S10: XPS Characterization of ssDNA adsorbed on Au(111). 26

S11: Spontaneous dissociation of DNA at is terminus. 28

S12: Cross-correlation analyses of conductance between adjacent plateaus. 30

S13: Determination of kinetic model by reaction plots. 32

S14: Details of simulation-based fitting of reaction plots. 34

S15: Kinetic effect of fluctuation in rate constants. 37

S16: Effect of surface modification on DNA hybridization. 40

References. 44

S1: Statistical analysis of plateaus in *I–t* traces.

For the statistical analysis, we constructed two-dimensional (2D) histograms of the plateaus observed in thousands of *I–t* traces (Fig. S1a). It was found that the height of the plateaus, which corresponds to the conductance of the molecular junction, are distributed around 1 mG_0 for all the sample surfaces (Fig. S1b), being consistent with the conductance value determined by the STM break-junction measurement of double-stranded DNA (dsDNA, see Section S2). The peak was fitted with Gaussian distribution, and the peak conductance and the full width at half maximum (FWHM) were listed in Table S1. The common peak conductance for all the three sample surfaces confirms that the individual plateaus correspond to the single DNA hybridized structure. On the other hand, the conductance distribution of the ordered sample surface shows much smaller FWHM than those of diluted and dense samples. We attribute the small conductance variation in the ordered sample to the restricted conformation or orientation in the densely-packed adlayer,[1] being consistent with the X-ray photoemission spectroscopy (XPS) study (see Section S10).

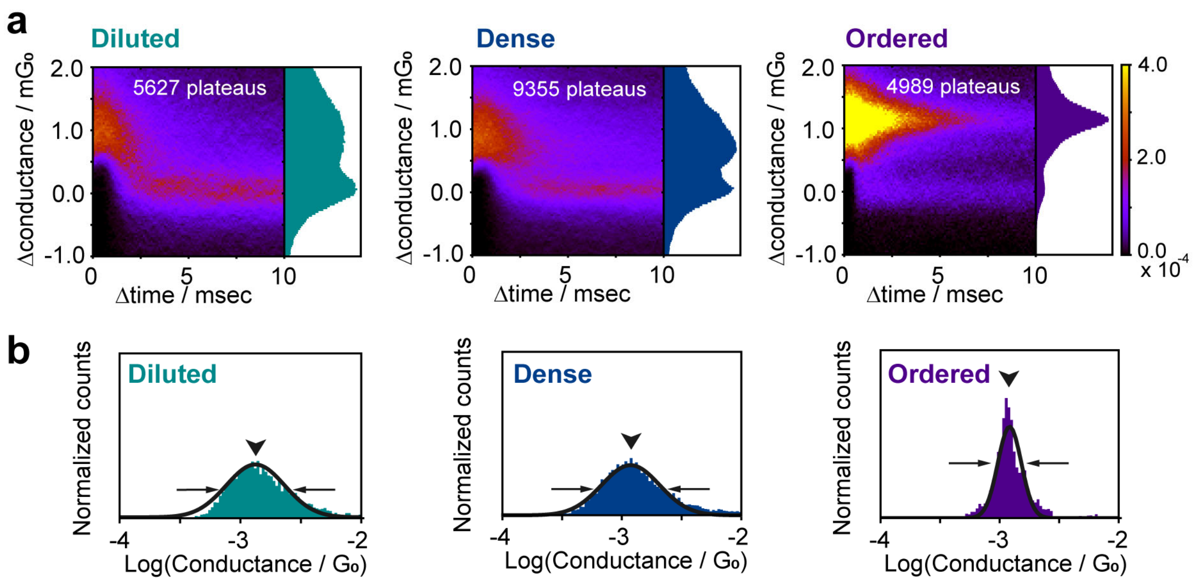

Figure S1. Analysis of plateaus in *I–t* traces. a, 2D histograms of plateaus. The origin of

the time (or conductance) axis is set to the onset of plateaus (or to the baseline of trace). **b,** Conductance histograms of plateaus. The histograms are constructed from 5627, 9355, and 4989 plateaus for diluted, dense, and ordered samples, respectively.

Sample	$\log(G/G_0)$	FWHM
Diluted	−2.8 ± 0.2	0.24 ± 0.04
Dense	−2.9 ± 0.1	0.27 ± 0.01
Ordered	−2.9 ± 0.2	0.15 ± 0.04

Table S1. List of the logarithm of conductance value and its full-width at half-maximum (FWHM).

S2: STM *G–z* measurements for single-molecule junction of dsDNA.

In addition to the *I–t* measurements reported in the main text, STM *G–z* measurements were performed to confirm the formation of the single-molecule junction of dsDNA. In *G–z* measurements, the STM tip was brought to the proximity of the substrate. After the dwell time for 0.2 s, the tip was pulled up to record the current (Fig. S2a). The 1D histogram constructed from these traces showed a single peak at $10^{-2.72}$ G_0 (Fig. S2b), being consistent with our earlier results.[2]

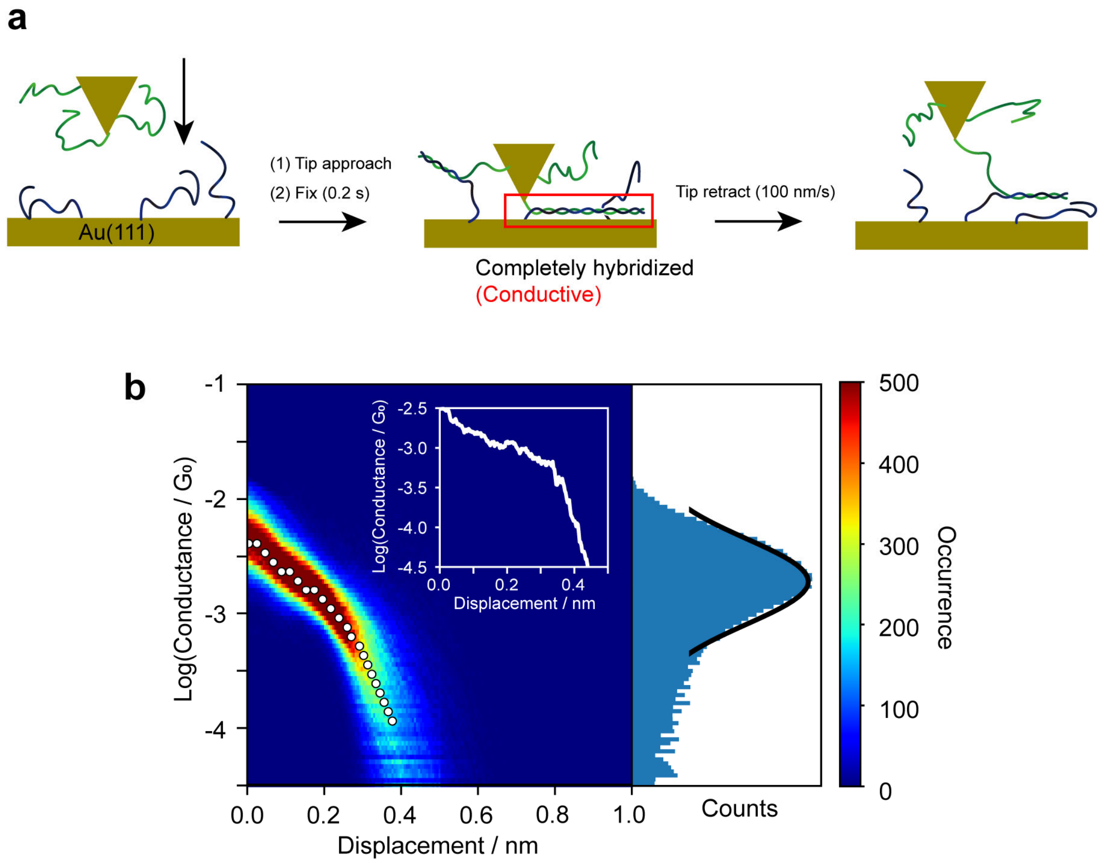

Figure S2. STM *G–z* measurement of hybridized DNA. a, Schematic illustration of *G–z* measurement. **b,** Histograms of *G–z* traces, constructed from 3618 traces. White dots in the 2D histogram represent the most probable conductance at each displacement. Inset shows a typical *G–z* trace.

The 2D histogram exhibited two regions where the logarithm of conductance decay with initial small slope (β_1) and subsequent large one (β_2). We previously demonstrated that the slower decay arises due to the formation of the single-molecule junction of dsDNA.[2] To statistically evaluate the β_1 and β_2 values, we fitted the individual *G*–*z* traces by two exponential functions. The point where the decay constant changed from β_1 to β_2 was determined according to the R^2 value of the fitting (Fig. S3a). The mean value of β_1 and β_2 was determined to be 3.3 and 12 nm^{-1}, respectively, from the histogram (Fig. S3b). This analysis also reveals the conductance value just before the breakdown of the single-molecule dsDNA junction (G_{change}). The mean value of G_{change} was found to be $10^{-3.23}$ G_0 (Fig. S3c).

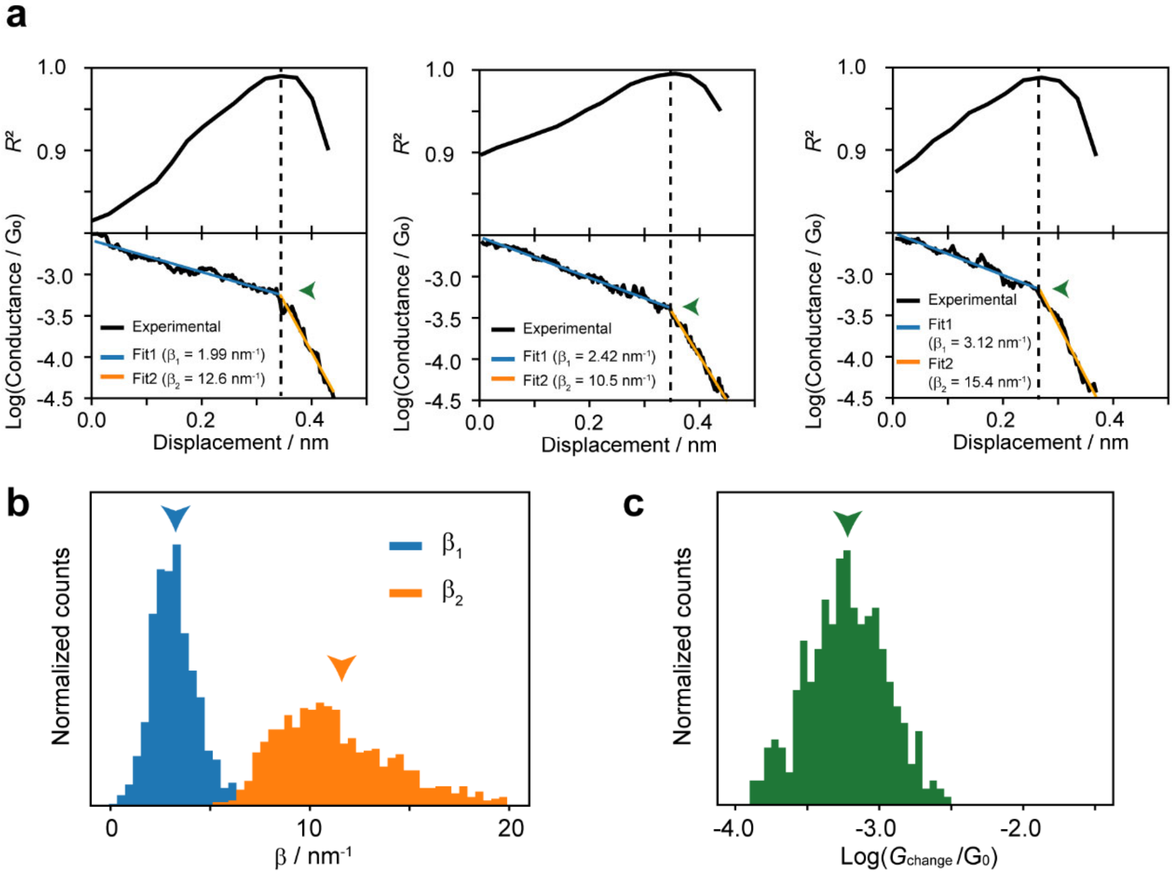

Figure S3. Analysis of junction breakdown in *G*–*z* traces a, Conductance traces fitted with two exponential functions. Top panels show fitting R^2 values. **b,** Histograms of β_1 and β_2 values. For the construction of the histogram, 3381 *G*–*z* traces were analyzed. **c,** Histogram of G_{change}.

S3: *I–t* measurements using non-complementary DNAs.

We performed additional *I–t* measurements as control experiments. The STM tip and substrate were modified with ssDNAs, and, as seen in the sequences in Fig. S4a, these strands were non-complementary to each other. The resulting *I–t* traces exhibited essentially no plateaus, and only rare transient current increases were found on the stable background current (Fig. S4b). These transient increases could be associated with unstable non-complementary base pairing between the ssDNAs on the tip and substrate. We thus conclude that the plateaus (Fig. 1b) arose because of the complementary duplex formed within the nanogap between the tip and substrate. Of note, there were partial complementarities with single base mismatches between the non-complementary ssDNAs used in the *I–t* measurements (Fig. S4a). Thus, the absence of plateaus in the control experiments further indicated that the partial hybridization and possible hairpin structures that arose from these partially matched sequences were insufficient to induce the plateaus.

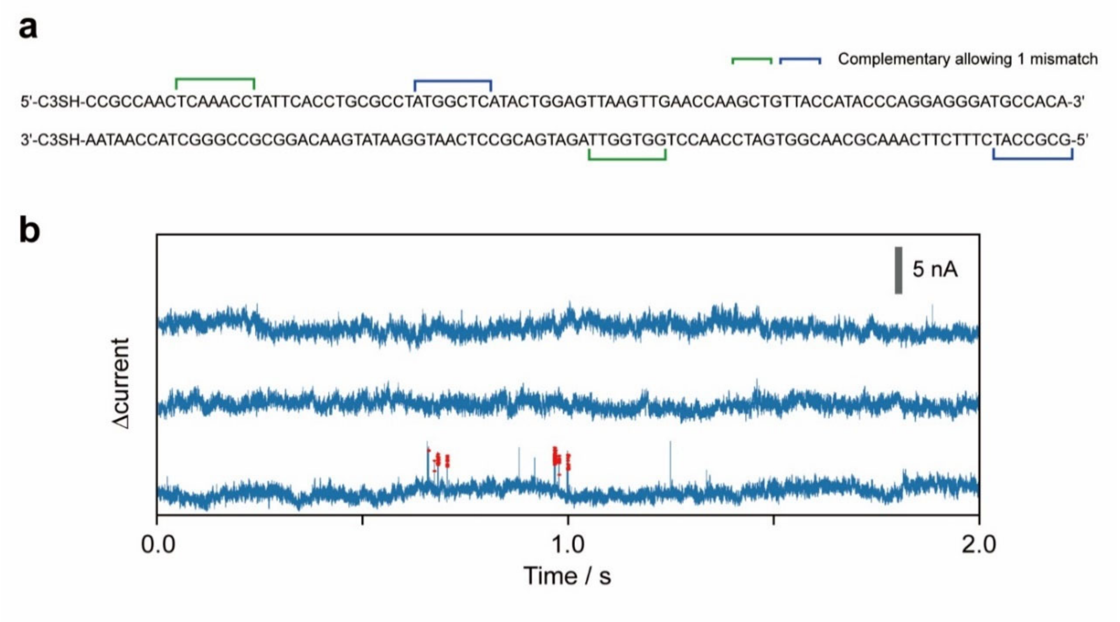

Figure S4. *I–t* measurements using non-complementary DNA. a, Sequences of the original 90-mer DNA and the non-complementary DNA. **b,** Typical *I–t* traces obtained with the non-complementary DNAs on the tip and substrate.

S4: Molecular dynamics study of DNA melting under mechanical forces.

Coarse-grained molecular dynamics (MD) simulation, using the CafeMol software[3] with 3SPN.2C model,[4,5] was performed to evaluate how the mechanical force exerted by the STM tip affects the melting process of the dsDNA in the molecular junction. One terminal of the DNA duplex was connected to two springs to model our STM experiments (Fig. S5a). These springs were displaced by certain distances to exert a mechanical force to the dsDNA terminal, and the intermolecular distance of the terminal base pair (d_{A1-B90}) were investigated (Fig. S5b).

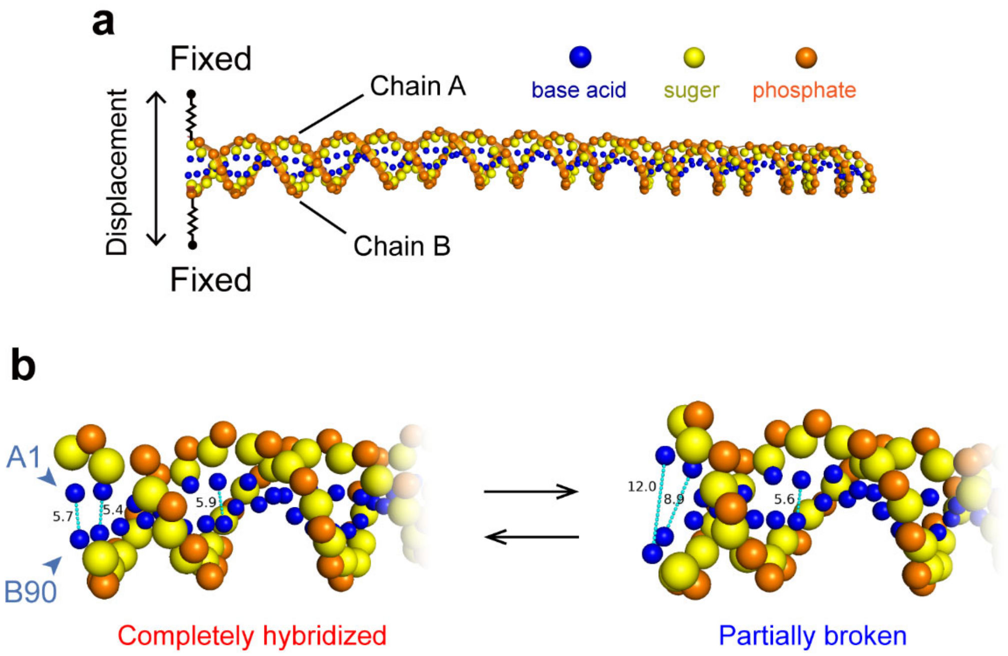

Figure S5. Coarse-grained MD calculation of DNA dehybridization. a, Schematic illustration of coarse-grained model of dsDNA. One terminus was tethered with two springs. The nitrogenous base, phosphate, and deoxyribose sugar were shown in blue, orange, and yellow, respectively. **b,** Typical coarse-grained structures of completely hybridized and partially broken duplexes. A few characteristic distances in Å between bases were shown.

Fig. S6a illustrates the time courses of $d_{A1–B90}$ with the displacement of the springs from 2.7 to 3.2 nm. One can clearly see that $d_{A1–B90}$ predominantly stayed around 0.6 nm, being the typical inter-base distance for the canonical base pairing,[6,7] with the displacement smaller than 3.0 nm. In addition, transient increases in $d_{A1–B90}$ were found, which indicates intermittent disruption of the base pairing. With the displacement larger than 3.0 nm, the $d_{A1–B90}$ increases occurred more frequently, due to the partial melting of dsDNA from the termini induced by mechanical forces.[8-11] The free energy landscape of the pairing between the bases A1 and B90 was estimated from the MD simulation on the basis of the weighted histogram analysis,[12] and we calculated the activation energy to break the base pairing as a function of the displacement of the springs (Fig. S6b). The resulting energy profile exhibited a constant activation energy of 7.7 kJ mol^{-1} when the displacement was smaller than 3.0 nm. The displacement-independent behavior demonstrates that the dsDNA spontaneously undergoes the partial melting through a thermally activated process. The decrease in the activation energy for the displacement larger than 3.0 nm indicates that the mechanical force assisted the dehybridization in this regime. We found that the simulated results quantitatively agree with the *I*–*t* measurement (Section S1), supporting the structural assignment of the intermediate species as the partially broken structure of dsDNA in the two-step model for the hybridization (Fig. 3c in the main text).

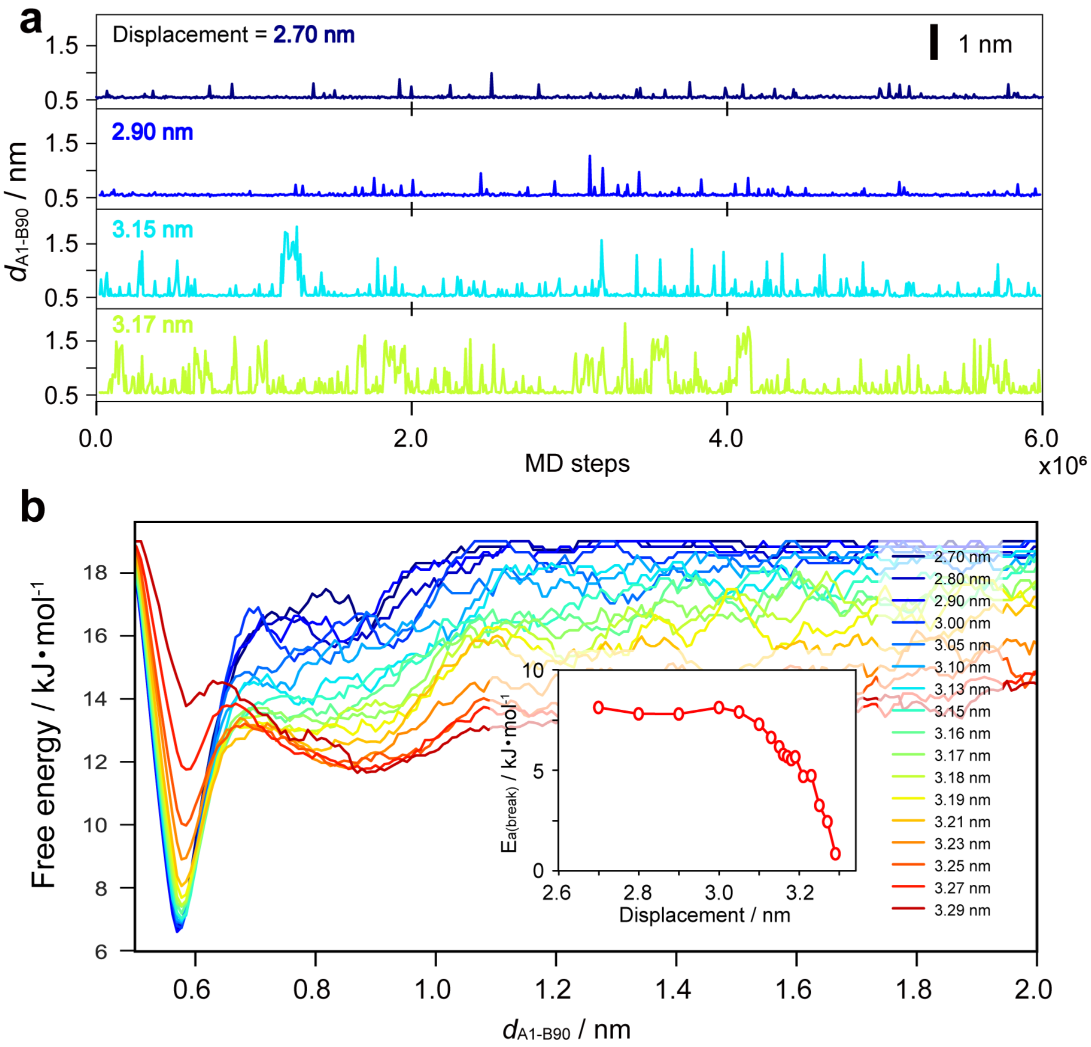

Figure S6. Base-pair dissociation under mechanical force. a, Time courses of the A1–B90 distances, $d_{A1–B90}$, with displacement of 2.70, 2.90, 3.15 and 3.17 nm. **b,** Energy landscape as a function of $d_{A1–B90}$ under different displacement ranging from 2.70 to 3.29 nm. Inset shows activation energy for the base-pair dissociation as a function of displacement.

We carried out similar analyses for the base pair of A90–B1 at the other terminal without the springs (Fig. S7a) in order to assess the effect of exerted forces on the activation energy. The time courses of the distance between A90 and B1 ($d_{A1–B90}$, Fig. S7b) still shows the occasional breaking of the base paring in spite of the absence of the mechanical perturbation. The activation energy

determined by the free energy landscape was found to be independent of the spring displacement for the whole range (Fig. S7c), indicating the thermally driven dissociation of this base pair. Importantly, the activation energy of 7.6 kJ mol^{-1} in Fig. S7c compares well with the constant activation energy of 7.7 kJ mol^{-1} to break the A1–B90 base pair for the displacement smaller than 3.0 nm. These energies support that the observation of the spontaneous process without mechanical perturbation can be achieved with the sufficiently narrow tip–substrate distance.

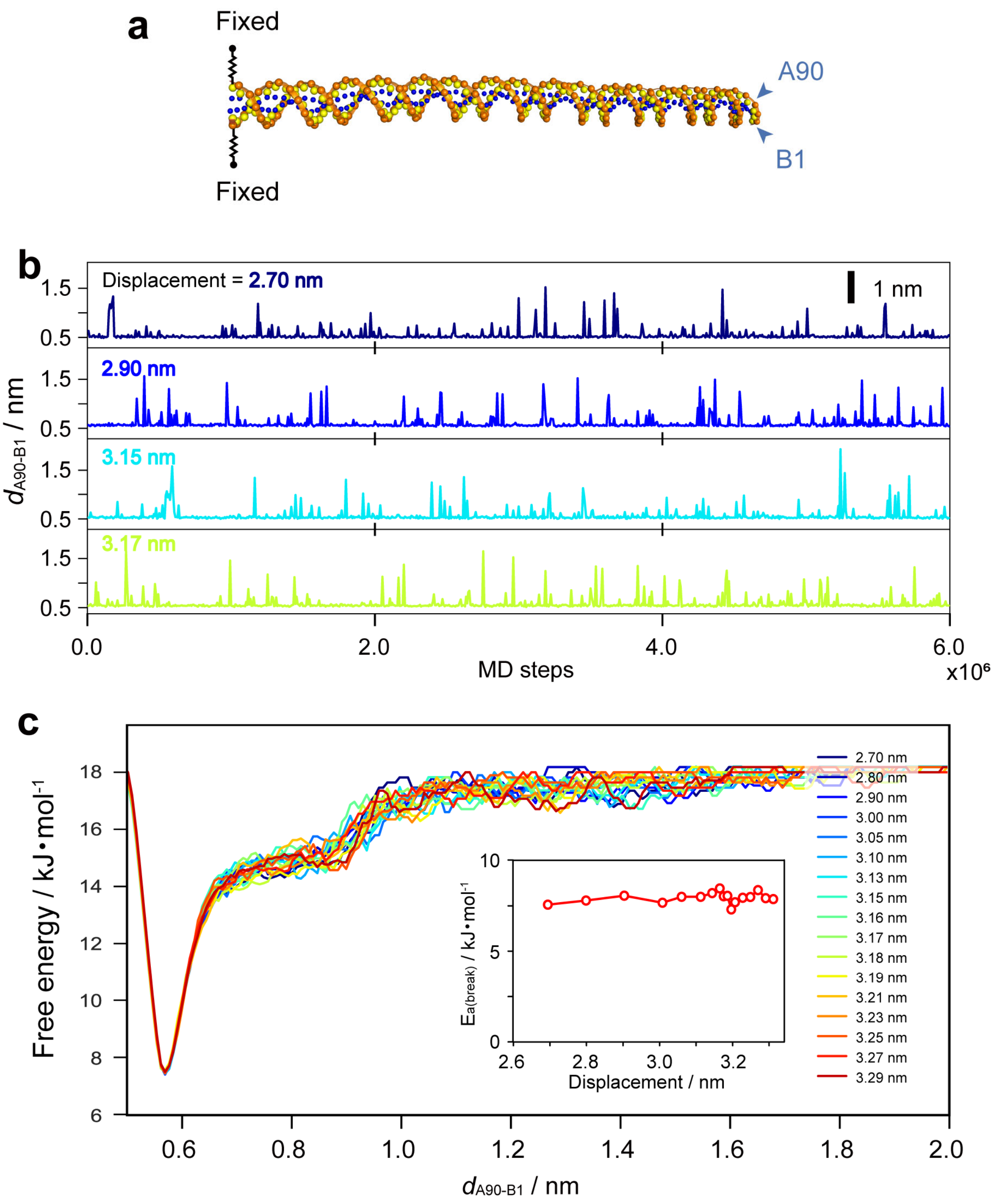

Figure S7. Dissociation of base pair at the free end of DNA. a, Coarse-grained DNA structure showing the base pair at the free end (blue arrowheads). **b,** Time courses of the A90–B1 distances, $d_{A90–B1}$, with displacement of 2.70, 2.90, 3.15 and 3.17 nm. **c,** Energy landscape as a function of $d_{A90–B1}$ under different displacement ranging from 2.70 to 3.29 nm. Inset shows activation energy for the base-pair dissociation as a function of displacement.

S5: Effect of mechanical force of tip on dehybridization kinetics.

To investigate the effect of the tip–substrate distance on the kinetics of the dsDNA hybridization in the junction structure, we evaluated the rate constant in the breaking process, k_b, of the junction under different set-point currents. The tip–substrate distance at each set-point current, Δz, was calculated relative to the distance at the current of 64 nA according to the equation $\Delta \log(G) = -\beta\Delta z$. The decay constant β was obtained from the *G–z* traces for the junctions without dsDNA. The *I–t* traces obtained with the set-point currents of 2–64 nA showed the high- and low-conducting states (Fig. S8a), as found in the main text. The conductance of the high-conducting states decreased exponentially as the tip–substrate distance increased (alternatively, as the set-point current decreased) as shown in Fig. S8c. The decay constant was determined to be 4.3 nm^{-1} from the plot, and this value reasonably agrees with the one found by the *G–z* study (3.3 nm^{-1}, see Fig. S3). The results support the assignment that the high-conducting state at every set-point current originates from the single-molecule junctions of the sample dsDNA.

The lifetimes of the high-conducting states were extracted from the *I–t* traces to construct the reaction plots (Fig. S8b), and the rate constant k_b of the dehybridization of dsDNA was determined for each set-point current as summarized in Fig. S8c. In the small tip–substrate distances, k_b was found to be constant but started to rapidly increase at the distance of 0.18 nm, which corresponds to the set-point current of 8 nA. The conductance at this distance ($10^{-3.2}$ G_0) was comparable to the conductance observed when the single-molecule dsDNA junction ruptured in the *G–z* measurements (see Fig. S3c). Taken together, the increase in k_b is attributed to the dsDNA dehybridization induced by the mechanical force of the probe tip, as previously reported for the single-molecule study of dsDNA[10] and alkanedithiol.[13] On the other hand, the constant k_b value found in the short-distance regime indicates that the mechanical forces have negligible effects on the dehybridization process at the set-point current larger than 8 nA. This conclusion is supported by the MD study described above (Section S4). The agreement with the simulation can be quantitatively confirmed with the activation energy E_a of

the dehybridization. The dependence of E_a on the tip–substrate distance was determined to be 3.3 kJ mol^{-1} nm^{-1} from Fig. S8c, and this experimental value reasonably agrees with the corresponding value determined by the MD simulation (3.6 kJ mol^{-1} nm^{-1}, Fig. S6b). On the basis of the results discussed above, all the experiments reported in the main text were carried out with the set-point of 16 nA to observe the spontaneous processes.

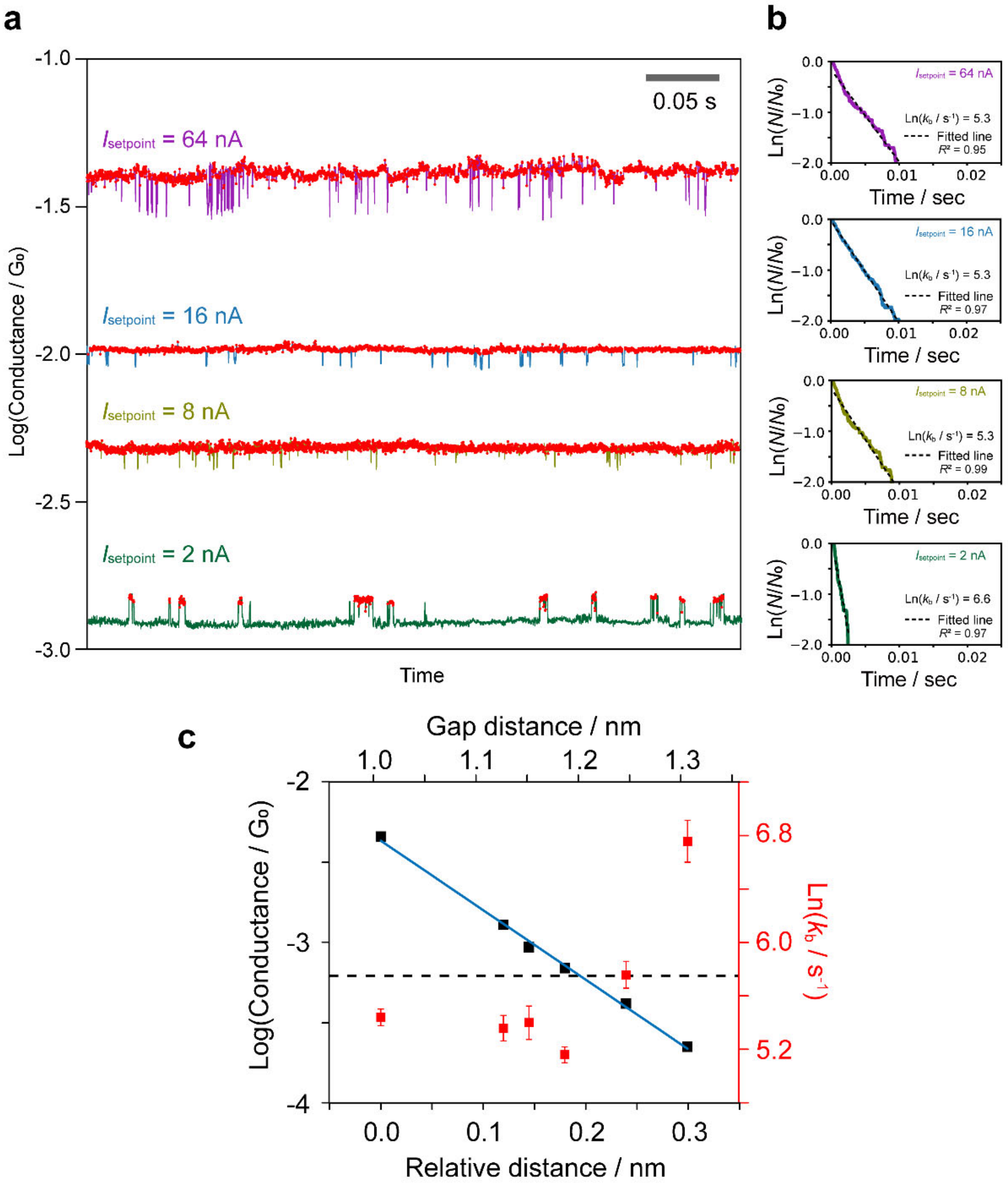

Figure S8. *I*–*t* measurements using different set-point currents. a, Typical *I*–*t* traces and

b, reaction plots for breaking process with set-point currents of 2.0, 8.0, 16.0, and 64.0 nA. Red dots indicate the conductance plateaus detected by ATA. **c,** Distance dependence of conductance and k_b, as determined using 1179, 2256, 4706, 1243, 712, and 1918 consecutive *I*–*t* traces for the set-point currents of 2, 4, 8, 12, 16, and 64 nA, respectively. The distance was estimated relative to the tip–substrate distance achieved with the set-point current of 64 nA. Black dotted line indicates the G_{change} value (see Section S2).

S6: Estimation of the tip–sample distance in the *I–t* measurement.

To estimate the gap distance between the STM tip and substrate in the *I–t* measurement, we analyzed the tip position dependence of the conductance.[14] Both the tip and substrate were modified with ssDNA, as in the *I–t* measurement reported in the main text. The conductance curves were recorded while the STM tip repeatedly approached the sample surface. The STM tip was stopped in each measurement when the conductance value exceeded $10^{-0.5}$ G_0 to avoid mechanical contact of the DNA tip with the substrate. Moreover, each measurement was performed after pulling up the tip by approximately 100 nm to ensure the dehybridization of DNA on the tip and substrate. A thousand conductance traces were collected to construct the 2D histogram, as shown in Fig. S9. The conductance exponentially increased with decreasing displacement as expected for the tunneling process. The average plot was extrapolated to the conductance value of 1 G_0, which arose upon the formation of Au atomic contacts and thus corresponds to a zero gap width. According to this procedure, the gap width of 0.69 nm was estimated for the set-point current of 16 nA, which was used during the *I–t* measurement, as described in the main text.

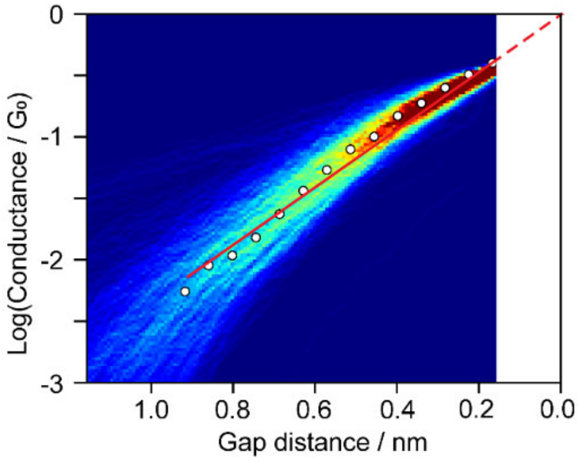

Figure S9. Determination of the gap width through *G–z* measurement during the approach process. a, 2D *G–z* histograms of the approach process. White circles indicate

the mode conductance value at respective gap widths. Red line shows the linear fit of the conductance values.

S7: Memory effect of rate constant in consecutive *I–t* measurement.

A joint-probability analysis, adapted from the analysis of single-molecule fluorescence measurements,[15-17] was carried out for evaluating temporal correlation in the rate constants for the DNA surface hybridization (Fig. S10a). The 2D joint probability $g(k_{b1}, k_{b2})$ denotes the probability to observe the rate constant k_{b2} after the rate constant k_{b1} in adjacent *I–t* traces (Fig. S10b). Another joint probability $h(k_{b1}, k_{b2})$ distribution was prepared for two *I–t* traces separated by 600 traces (Fig. S10c). In $h(k_{b1}, k_{b2})$, the two rate constants, k_{b1} and k_{b2}, almost lose the mutual correlation because of the large separation in-between. A difference distribution in Fig. S10d, $g(k_{b1}, k_{b2}) - h(k_{b1}, k_{b2})$, highlights the memory effect. The difference distribution exhibited large positive diagonal counts, indicating the presence of correlation between k_{b1} and k_{b2}. The strong correlation between the adjacent rate constants supports the reproducible formation of the molecular junction of the DNA involving the same strands during a few consecutive measurements.

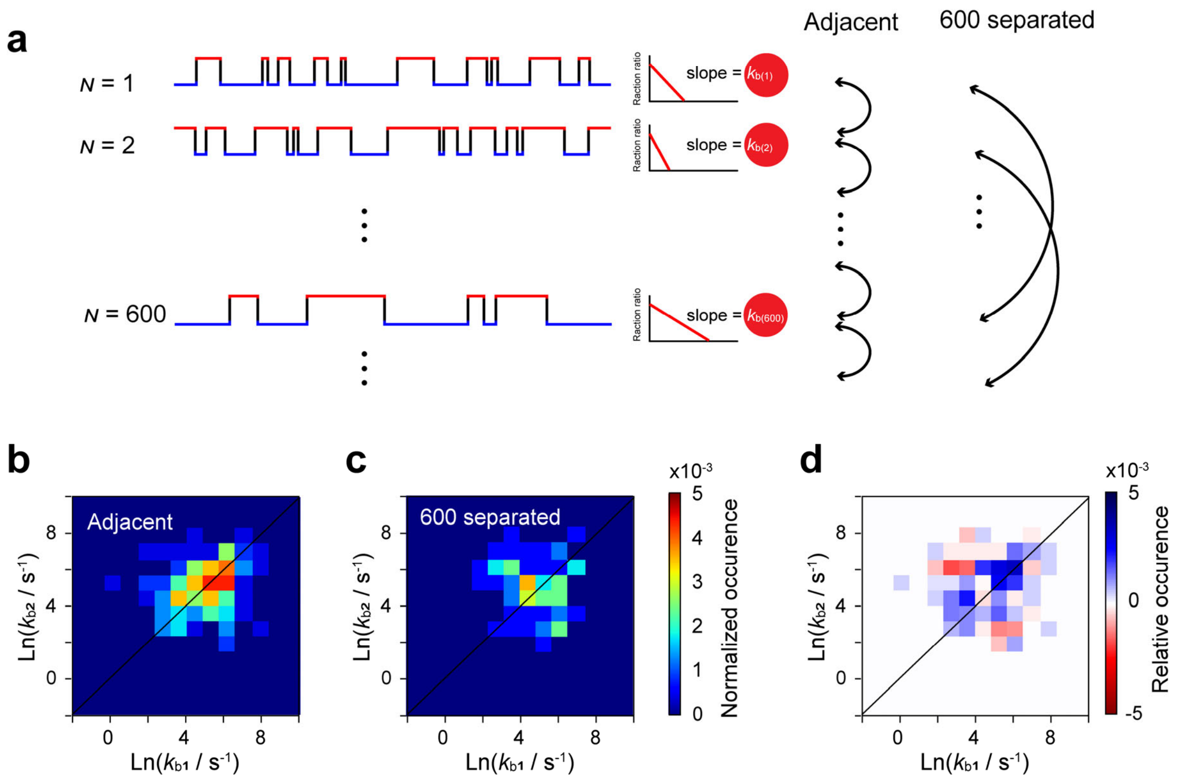

Figure S10. 2D joint-probability distribution of rate constant. a, Schematic illustration of the joint-probability analysis. The joint-probability distribution of **b**, adjacent dwell lengths, $g(k_{b1}, k_{b2})$, and **c,** dwell lengths separated by 600 traces, $h(k_{b1}, k_{b2})$. **d,** The difference histogram, $g(k_{b1}, k_{b2}) - h(k_{b1}, k_{b2})$.

To further investigate the timescale of k_b correlation, we calculated auto-correlation function of the rate constant $C_{kb}(n) = \langle k_b(0)\ k_b(\mathrm{n})\rangle / \langle k_b^2\rangle$, where n is the separation between I–t traces (Fig. S11). The $C_{kb}(n)$ has been known to follow a stretched exponential function, $C_{kb}(n) = C_{kb}(0)\exp[-(t/t_0)^{\beta}]$. For the resulting auto-correlation function, $\beta = 0.52$ and $t_0 = 2.4$, which is equivalent to 6.0 s, were found. During this time period, the tip drifts for a few Å according to the drift velocity estimated in Section S8, indicating that the variation of the rate constants were due to the difference of the DNA strands in the molecular junction. A similar observation in the fluctuation in the rate constant is reported in single-molecule studies by an STM break-junction technique, where broadly distributed rate constants of electron transfer arises due to the difference in the molecule-electrode geometry or molecular conformations.[18]

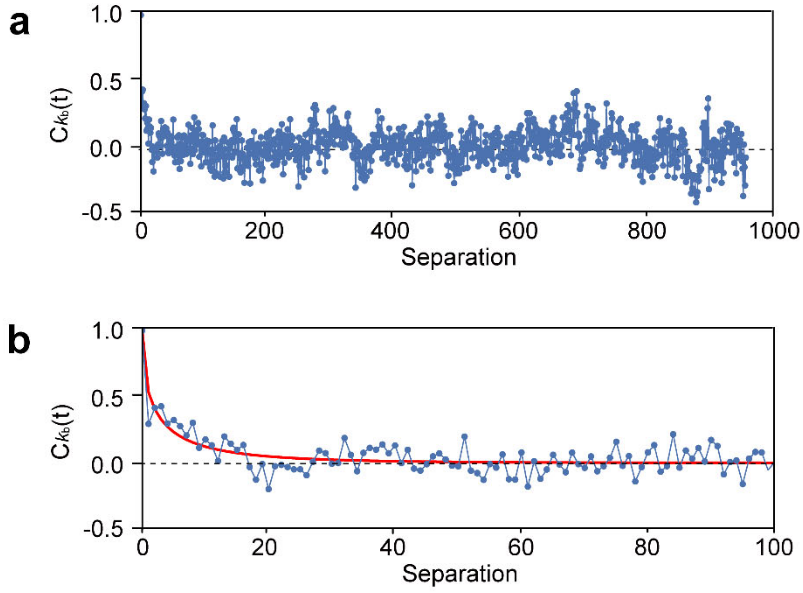

Figure S11. Auto-correlation function of rate constant in breaking reaction. a, Overview and **b,** enlarged view of the auto-correlation function. Red line shows the fitted stretched exponential function, $C_{kb}(n) = C_{kb}(0)\exp[-(t/t_0)^{\beta}]$ with $\beta = 0.52$ and $t_0 = 2.4$.

S8: Evaluation of STM tip drift

To evaluate the effect of the drift of the STM tip on the *I*–*t* measurements, we performed continuous STM imaging of an unmodified Au(111) surface (Fig. S12a). One of the structures characteristic to the Au(111) surface was traced during the consecutive images to calculate the drift distance. We found that the distance linearly depended on time, and the drift velocity of 0.035 nm s^{-1} was determined for our experiments (Fig. S12b). Since, in the *I*–*t* measurements, a single trace was acquired in 2.5 s, the drift distance within the trace was below 1 Å. We thus conclude that the single-molecule junctions detected in a particular *I*–*t* trace were formed at atomically the same location. However, a dataset of the *I*–*t* measurement contains thousands of traces. The drift in the direction perpendicular to the substrate surface was compensated before each measurement, but no correction was made in the parallel direction. Thus, the statistical analysis reflects the variation of the surface structure of the adsorbed DNAs in a sub-micrometer scale along the surface.

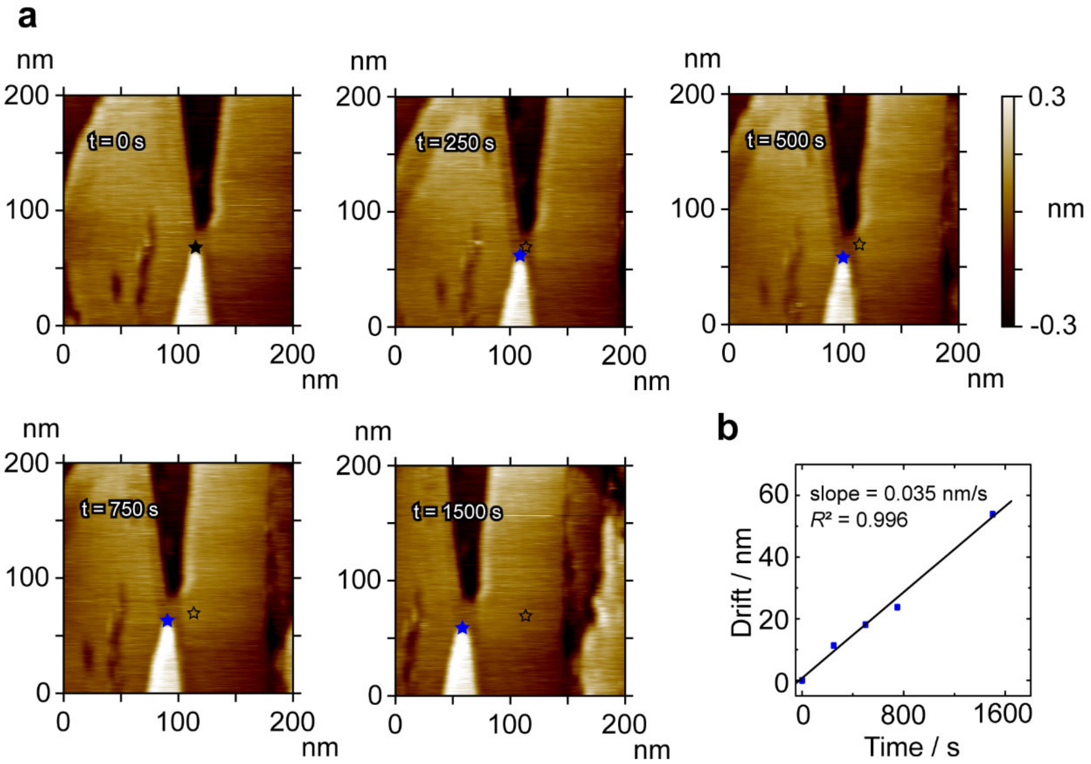

Figure S12. The time dependence of the STM tip drifts. a, STM images of Au(111) surface continuously acquired over 1500 s. Bias voltage, 20 mV; set-point current 2 nA. The black-filled and blue stars indicate the initial and drifted reference points of the Au(111) structure, respectively. The empty black starts represent the coordinate of the initial reference point. **b,** Time dependence of drift distance, as evaluated by the reference points.

S9: Electrochemical measurements of the DNA-modified gold surfaces.

Electrochemical measurements were used to estimate the surface coverage of the DNA on the Au substrate. First, the effective surface area of the substrate surface was determined by the cyclic voltammogram (CV) obtained using the bare Au(111) substrate in 0.05 M H_2SO_4 solution (Fig. S13a). The sharp anodic peak at 1.3 V and the cathodic peak at 0.9 V (vs. Ag/AgCl) are associated with adsorption and desorption, respectively, of the oxygen on the Au(111) surface.[19] The cathodic current was used to calculate the effective surface area. Next, CVs were recorded in a 0.1 M KOH solution using Au(111) substrates modified with single-stranded DNA (ssDNA) at coverages corresponding to the diluted, dense, and ordered samples (Fig. S13b). The resulting CVs exhibited the typical anodic peak at 0.8 V (Fig. S13b) owing to the reductive desorption of thiolate groups on ssDNA. The charge amounts of the diluted, dense, and ordered samples were calculated to be 1.4×10^{-6}, 1.7×10^{-6}, and 5.2×10^{-6} C cm^{-2}, respectively. Because the thiol reductive desorption is a one-electron process, the charge can be translated into DNA coverage, leading to coverages of 7.4×10^{12}, 9.2×10^{12}, and 2.7×10^{13} molecules cm^{-2} for the diluted, dense, and ordered samples, respectively. The XPS investigation revealed that the ssDNA of the ordered sample adopted a free-standing configuration, whereas the molecules of the diluted and dense samples lied flat on the Au substrate surface (Section S10). Combining the results of the electrochemical and XPS studies showed that the structural change from the flat-lying to the free-standing configurations occurred when the surface coverage of the ssDNA became larger than approximately 10^{13} molecules cm^{-2}, which was consistent with the literature.[20,21]

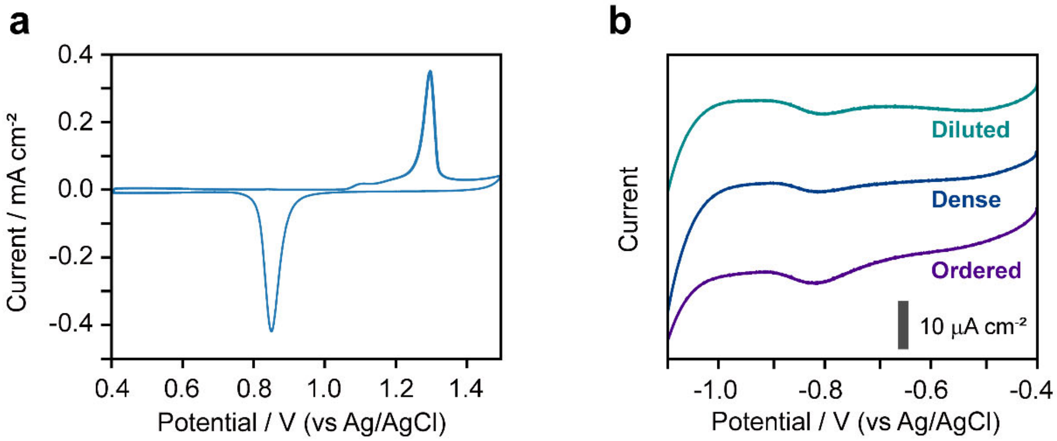

Figure S13. Electrochemical characterization of ssDNA on Au(111) surfaces. a, Cyclic voltammogram of unmodified Au(111) surface in 0.1 M H_2SO_4 solution. Scan rate, 50 mV s^{-1}. **b,** Reductive desorption of ssDNA/Au(111) for the diluted, dense, and ordered samples in 0.1 M NaOH solution. Scan rate, 50 mV s^{-1}.

S10: XPS Characterization of ssDNA adsorbed on Au(111)

The adsorption of single-stranded DNA (ssDNA) for the three sample surfaces used in the present study was investigated by XPS. These sample surfaces were prepared by the immersion of the Au substrate in either the 20 nM or 1 μM ssDNA solutions for 1 min or 2 h (see Experimental). The XP spectra of the N 1s region were shown in Fig. S14a. In all the samples, the N 1s peak was observed at around 401.0 eV, which is a typical value for a free nitrogen in the DNA bases.[22] For the comparison of the coverages of immobilized DNA among the three sample surfaces, we analyzed the peak intensity ratio of the N 1s to Au $4f_{7/2}$ (Fig. S14b). This ratio was found to increase with the immersion time and concentration of the ssDNA solution employed in the sample preparation. Another peak around the low binding energy was observed at ~399 eV. This component is attributed to a chemisorbed nitrogen, suggesting a strong interaction between the DNA bases and the Au surface.[22] The peak was strongly suppressed for the sample with the highest coverage (Fig. S14c). The decrease in the chemisorbed DNA bases indicates the rearrangement of the adsorbed structures of ssDNA. The ssDNA molecules initially lied flat on the Au surface when the ssDNA coverage was low. Upon the coverage increase, the ssDNA molecules adopt a free-standing configuration.[23,24] Similar rearrangement of adsorbed ssDNA was reported by XPS, IRAS, and NEXAFS studies.[22,25,26] The coverage of ssDNA and the adlayer structures for the three kinds of sample surface (diluted, dense, and ordered), as inferred from the XPS studies, were schematically summarized in Fig. S14d.

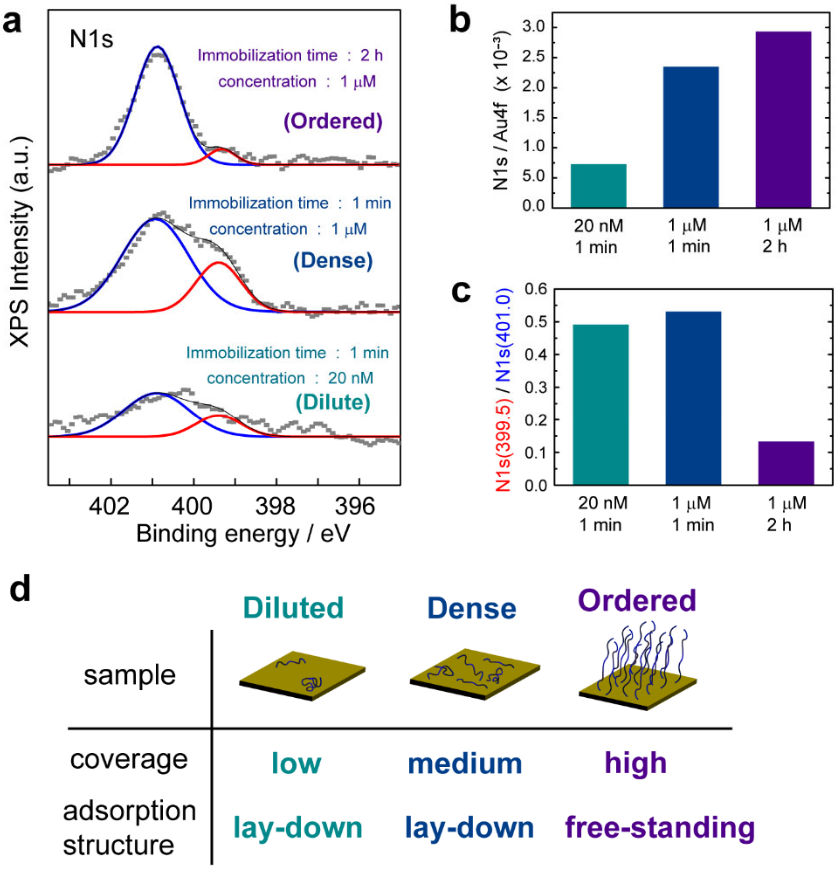

Figure S14. XPS analyses of DNA adlayers on Au(111). a, XP spectra of N1s region for three surface types. **b,** N1s peak intensity, normalized by that of Au4f, for three sample surfaces. **c,** The peak intensity ratios N1s(399.5 eV) / N1s(400.5 eV). **d,** Schematic illustration of surface structures of three sample surfaces.

S11: Spontaneous dissociation of DNA at its terminus.

The rate constant of the DNA dehybridization, k_b, allowed us to determine the activation energy, $\Delta G^{\ddagger}$, to be 61 to 62 kJ mol^{-1}. On the basis of $\Delta G^{\ddagger}$, we can further discuss the initial process of DNA dehybridization. It is known that $\Delta G^{\ddagger}$ depends on the number of unpaired bases, N_{bp}, at the early stage of dehybridization. This relationship is approximated as $\Delta G^{\ddagger} = \Delta G_0^{\ddagger} + N_{bp}\Delta g$, where $\Delta G_0^{\ddagger}$ and Δg are the length-independent term and average free energy per base pair, respectively.[27] The calculated value of $\Delta G^{\ddagger}$ indicates that dehybridization initiates with the separation of approximately seven base pairs. However, the exact number of dissociated base pairs should vary depending on the experimental conditions. We supplemented the aforementioned discussion with MD calculations of dsDNA. The MD simulations, as detailed in S4, were analyzed focusing on the terminal 30 base pairs by monitoring their base–base distances (Fig. S15a). The threshold distance for the dissociation of the base pairing was set at a distance of 6.1 Å. The time course of the number of undissociated base pairs is shown in Fig. S15b. A few base pairs undergo spontaneous dissociation. Figure S15c shows the histogram of the number of dissociated base pairs. The average number of dissociated pairs was calculated to be 2.7. Thus, the estimations based on the activation energy and MD simulations suggest that the dehybridization process is initiated from the dissociations of a few terminal base pairs.

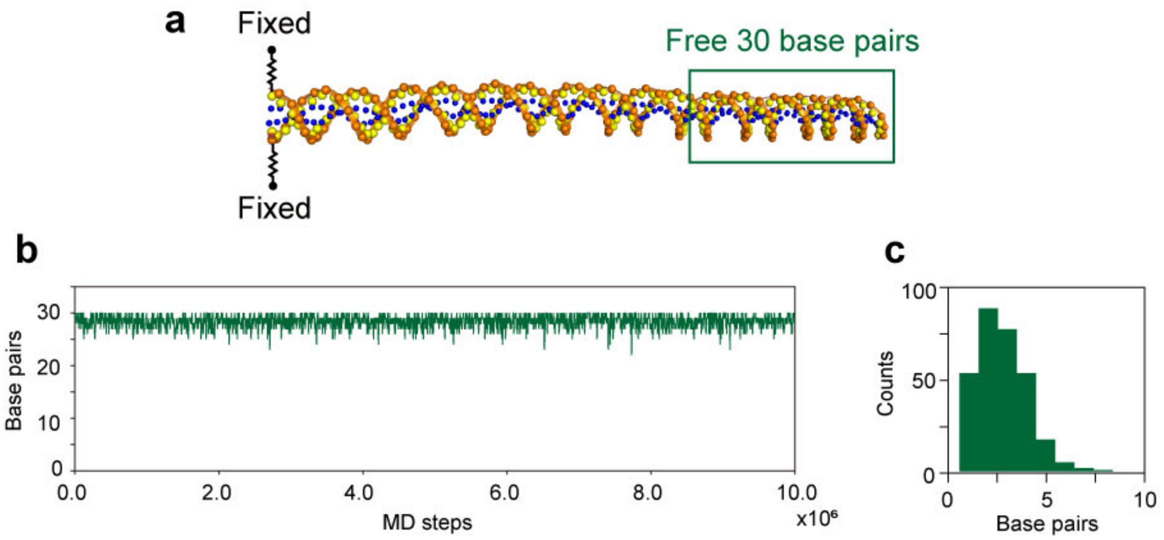

Figure S15. Analysis of base paring during MD simulations. a, Coarse-grained DNA structure and the 30 base pair at the free end (green square). **b,** Time courses of the number of the undissociated base pairs. **c,** Histogram of number of dissociated base pairs.

S12: Cross-correlation analyses of conductance between adjacent plateaus.

We evaluated the similarity between the adjacent conductance plateaus that occurred before and after the certain dwell time by cross-correlation analyses (Fig. S16a). The scatter plot of the cross-correlation against the dwell time for the whole *I–t* measurements was shown in Fig. S16b. Two distributions were found by clustering the plot by the fuzzy c-means: one with the low cross-correlation and the long dwell time and another with the high cross-correlation and the short dwell time. The presence of the two subgroups is consistent with the kinetic model that involves the two broken states (Fig. 3c in the main text). To further investigate the two distributions, the same scatter plots were constructed for the three sample surfaces, i.e., the diluted, dense, and ordered samples (Fig. S16c). The plots for the diluted and dense sample surfaces show a similar occurrence of the two distributions. In contrast, the distribution with the high cross-correlation and the short dwell time prevails for the ordered sample. We attribute this distribution to the recovery process where the dsDNA is regenerated from the partially melted DNA strands (Fig. 3c in the main text). The other distribution, showing the low cross-correlation and the long dwell time, corresponds to the hybridization process initiated with the fully dissociated ssDNAs. The decreased cross-correlation after the long dwell time could be caused by the conformational change of the molecular junction via the surface adsorption. An alternative explanation could be the participation of other DNA strands under different local surface environments in the molecular junctions formed before and after the long dwell times.

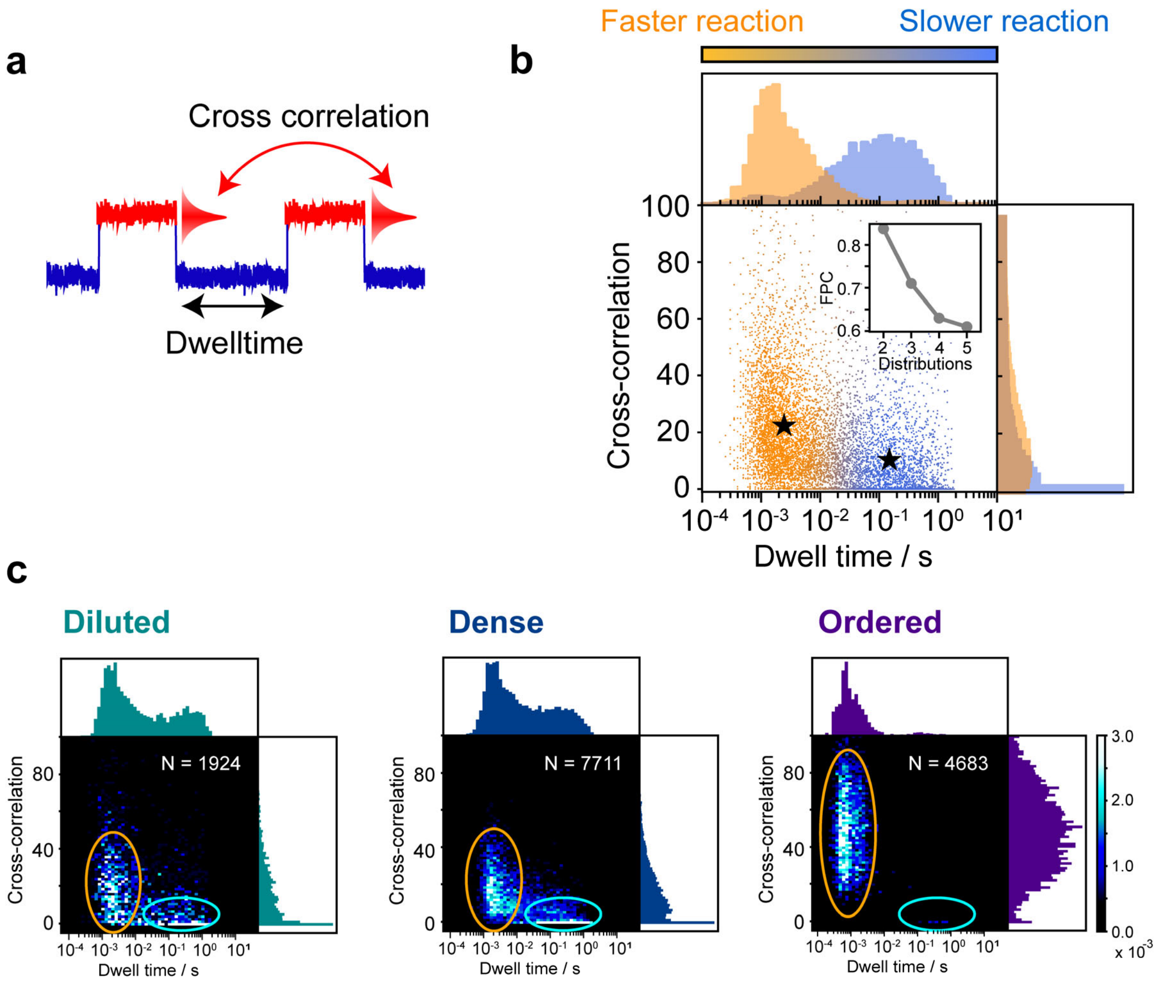

Figure S16. Cross-correlation analysis of adjacent plateaus in *I*–*t* traces. a, Schematic illustration of cross-correlation analysis. **b,** Scatter plot of cross-correlation and dwell time. Stars indicate two subgroups clustered by fuzzy c-means. Inset shows fuzzy partition coefficient (FPC). **c,** 2D histograms of cross-correlation and dwell time for three surface types. Yellow and cyan circles indicate two subgroups.

S13: Determination of kinetic model by reaction plots.

To determine the kinetic model for the hybridization observed by the *I–t* measurements, possible models were considered. A few examples of the model were illustrated in Fig. S17a: Type I contains independent multiple elementary reactions, Type II consists of multiple broken DNA structures that result in a common hybridized structure, and Type III is a sequential reaction involving two broken structures. We anticipate a first-order kinetics for each independent elementary reaction in the kinetic models. In contrast, the reactions affording a common product from multiple processes obey non-first-order kinetics. Different combinations of the reaction orders for the hybridization and de-hybridization reactions are expected depending on the kinetic models as summarized in Fig. S17a. We thus examined reaction order from the reaction plots to determine the most plausible kinetic model. The reaction plots were fitted by the linear regression model under the assumption of the first-order kinetics, and apparent rate constants for the hybridization (k_f) and dehybridization (k_b) were derived. The scatter plot of k_f against k_b clearly shows two subgroups of the pair of the rate constant (Fig. S17b). While the difference in the k_b values between the two subgroups was negligible, the k_f in one subgroup was larger than the other.

Next, the validity of the assumption of the first-order kinetics was assessed by the coefficient of determination of the fitting procedures (R_f^2 and R_b^2 for the hybridization and de-hybridization, respectively; Fig. S17c). The R_b^2 was found to be around 1.0, showing the first-order kinetics of the de-hybridization reaction. On the other hand, small R_f^2 values were obtained for a significant amount of the reaction plots. These coefficients indicate the non-first-order reaction for the hybridization reaction. Collectively, it was concluded that the Type III model (Fig. S17a) is the most probable for the present observation by the *I–t* measurements. Close inspection of the R_f^2 distribution corroborates our assignment. The R_f^2 approaches unity for the large k_f, while the coefficient becomes small for the small k_f (Fig. S17c). The Type III model well explains this behavior: the hybridization from the first broken state (Broken 1 in Fig. S17a) quickly, and thus with the large k_f, proceeds via the first-order

reaction. However, the second broken state (Broken 2) tentatively yields the Broken 1 state and then produced the hybridized state. This reaction pathway exhibits the small rate constant and the non-first-order kinetics.

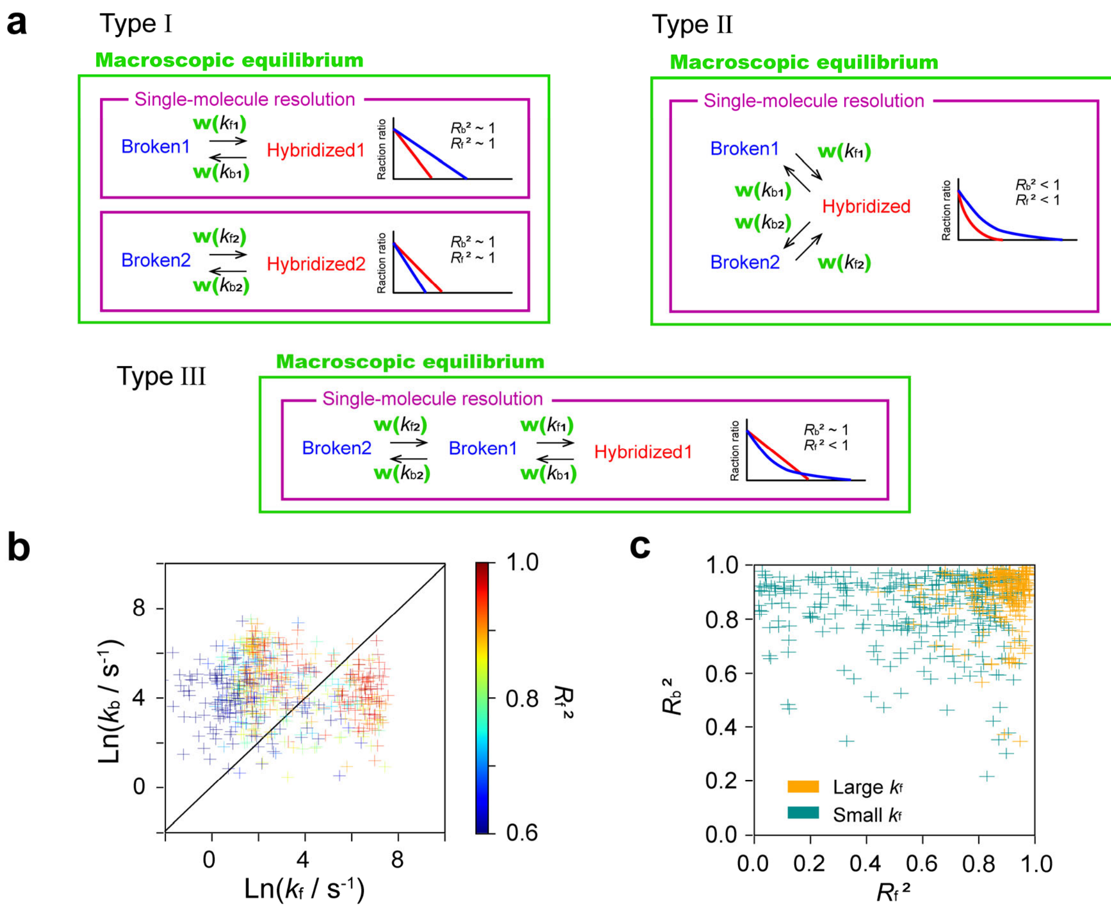

Figure S17. Possible kinetic models. a, Schematic illustration possible kinetic models, along with expected reaction plots. **b,** Scatter plot of the logarithm of rate constants for breaking and hybridization processes. Each data point is color-coded by R_f^2 value. **c,** Scatter plot of R_f^2 and R_b^2 values. The orange and blue points correspond to the two subgroups found in Fig. S16. The plot was constructed using 546 *I*–*t* traces.

S14: Details of simulation-based fitting of reaction plots.

The reaction plots are largely affected by the fluctuation of the rate constants due to the different microscopic environments of the adsorbed DNAs. For accurate estimation of these constants, simulation-based parameter fitting was applied to the analyses of the averaged reaction plots (Fig. S18). In this protocol, log-normal distributions of the rate constants k_i (i = 1–3), i.e., Gaussian distributions of $\ln(k_i)$, were fitted to the experimental results via their mean values $\mu(k_i)$ and distribution width $\sigma(k_i)$. First, initial distributions $w(k_i)$ were assumed, and 1000 reaction plots were numerically generated using k_i randomly sampled from $w(k_i)$ according to the following kinetic equations.

$$\frac{dN_{\mathrm{D}}}{dt} = -k_1 N_{\mathrm{D}} + k_2 N_{\mathrm{M}}$$

$$\frac{dN_{\mathrm{M}}}{dt} = k_1 N_{\mathrm{D}} - k_2 N_{\mathrm{D}} - k_3 N_{\mathrm{M}}$$

$$\frac{dN_{\mathrm{H}}}{dt} = k_3 N_{\mathrm{M}}$$

In these equations, N_{H}, N_{M}, and N_{D} are the populations of the fully hybridized, partially melted, and fully dissociated DNAs, respectively. Importantly, the present measurement protocol using the molecular tip allows us to eliminate consideration of the ssDNA on the tip from the kinetic equations. This is because the strands on the tip was held in close proximity to the substrate and, consequently, their population was kept constant during the measurements. The averaged plots were calculated from the reaction plots and compared with the experimental counterpart (Fig. 4a in the main text). This procedure was repeated until consistency between the simulated and experimental plots was met. Genetic algorithm was utilized in the modification of $\mu(k_i)$ and $\sigma(k_i)$ during the optimization.

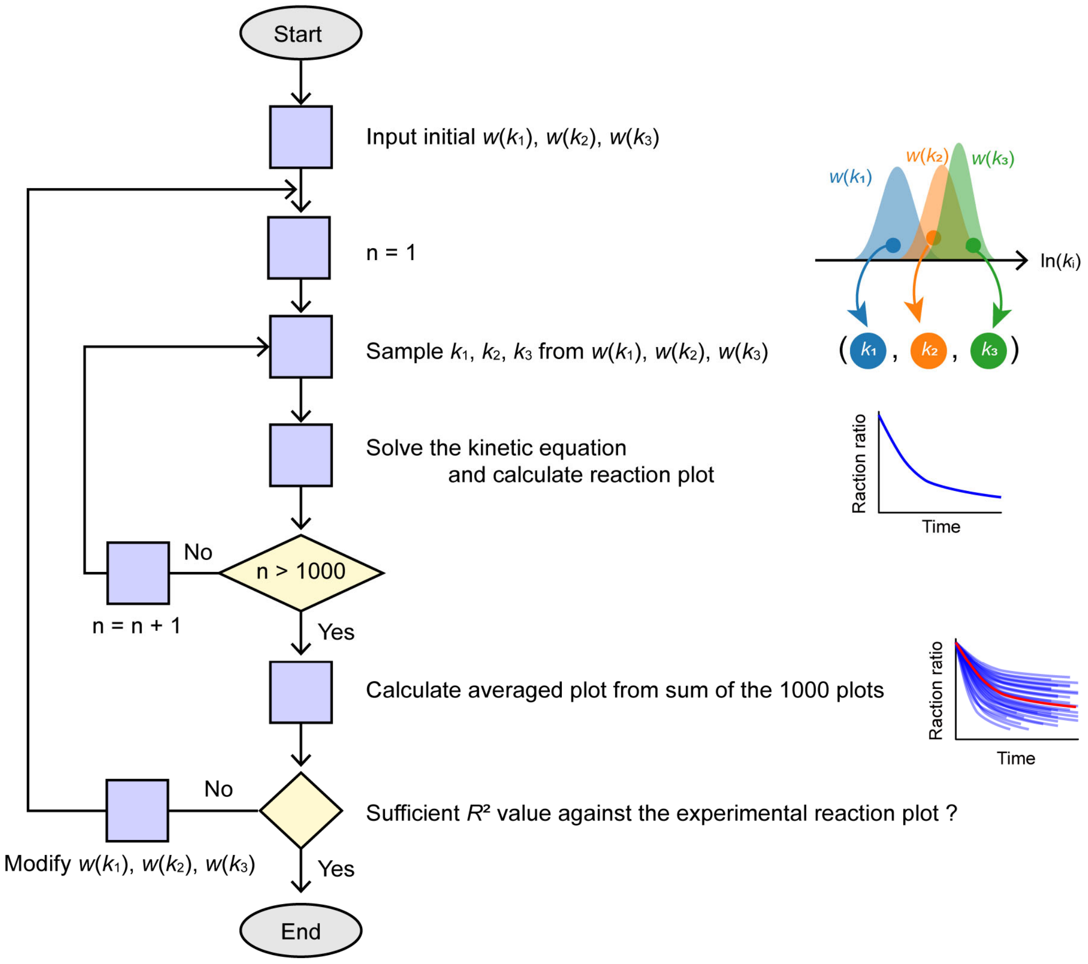

Figure S18. Diagram of simulation-based fitting protocol. Log-normal distributions of rate constant were assumed and optimized via comparison between simulated and experimental reaction plots.

We further assessed the validity of the simulation-based fitting by a correlation coefficient between the experimental and simulated reaction plots (Fig. 4a and 4b in the main text, respectively). The correlation coefficient was calculated by

$$C_{coeff} = \frac{\sum_{x,y} \Delta I_{1(x,y)} \Delta I_{2(x,y)}}{\sqrt{\sum_{x,y} \Delta I_{1(x,y)}^2 \sum_{x,y} \Delta I_{2(x,y)}^2}}$$

, where

$$\Delta T_{\mathrm{i}(x,y)} = I_{\mathrm{i}(x,y)} - \frac{\sum_{x,y} I_{\mathrm{i}(x,y)}}{wh}$$

and $I_{\mathrm{i}(x,y)}$ is an intensity of the data point at (x, y) in the reaction plot. And w and h are the total number of the data points in the vertical and lateral directions, respectively. The matrix of the calculated C_{coeff} (Fig. S19) shows that only the diagonal elements are nearly unity. The results prove that the simulation-based fitting reproduces the experimental results, which ensures the correct estimation of the rate constants.

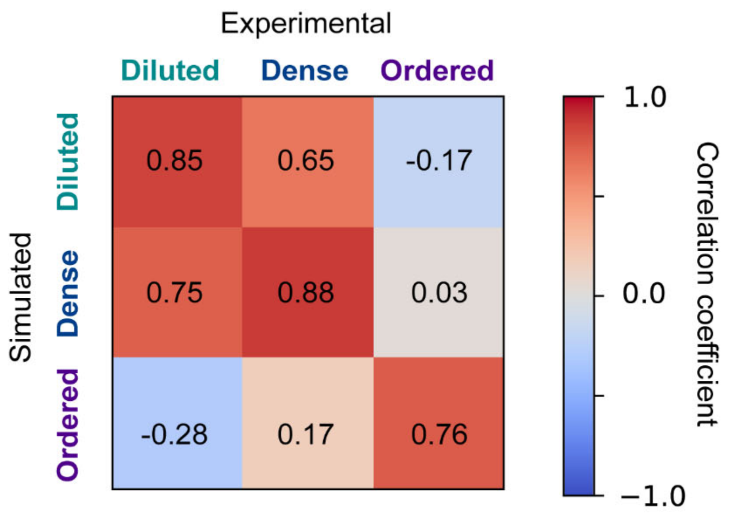

Figure S19. The matrix of correlation coefficients between experimental and simulated reaction plots. Experimental and simulated reaction plots shown in Fig. 4a and 4b, respectively, in the main text were used to calculate the coefficients.

S15: Kinetic effect of fluctuation in rate constants.

For the breaking process of the hybridized DNA, inferred from the lifetime, we observed well-defined linearity in the reaction plots constructed from the single *I–t* traces (Fig. 2a in the main text), which indicates the first-order kinetics. However, the plot constructed from thousands of *I–t* traces lost the linearity, and this behavior is attributed to the fluctuation in the rate constant. Similar observations have been reported in dynamic disorder of enzymatic reactions.[15,16] A linear regression analysis of the latter plot is clearly inappropriate for the estimation of the rate constant (Fig. S20a). The accurate estimation requires the measurements that eliminate the origin of the fluctuation in the rate constants by, e.g., acquiring data under the spatially and temporally confined circumstances. Conventional ensemble measurements are inapplicable, but the present *I–t* studies fulfill the requirement and indeed succeeded in the precise determination of the distribution of the rate constants, as discussed in the main text.

Regarding the hybridization reaction, accessed by the dwell time, the analysis of individual reaction plot indicated that the sequential two-step process is the most relevant kinetic model: the fast process to regenerate the dsDNA from the partially melted structure and the slow process to form the partially melted structure from the fully dissociated ssDNAs. The fast and slow processes dominate the reaction plot in the short- and long-time regimes, respectively. We demonstrated the successful estimation of the distribution of the rate constants from the reaction plot in the main text (Fig. 4). It was also found that the reaction plots calculated with rate constants without considering the fluctuation, i.e., without the distribution of the constants, cannot reproduce the experimental reaction plots (Fig. S20b). These results emphasize the importance of the fluctuation in the rate constants in describing kinetic behaviors at the single-molecule level.

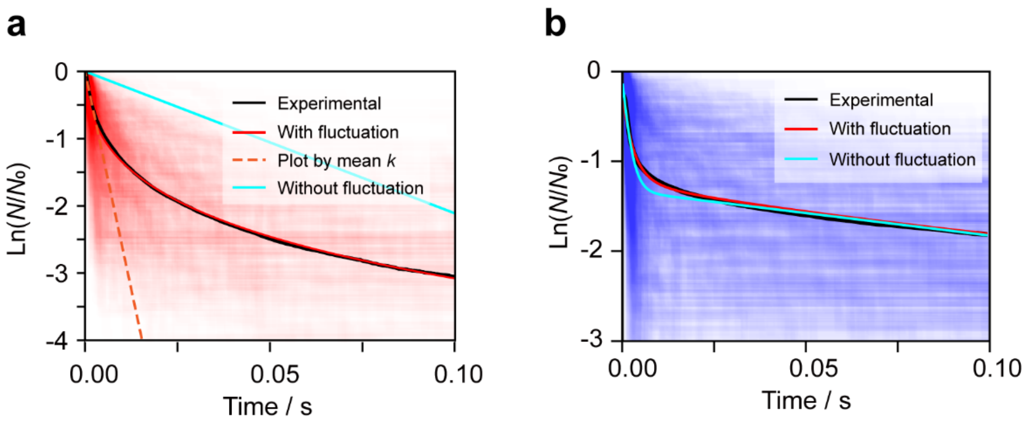

Figure S20. Estimation of rate constant from reaction plot. Reaction plot for breaking (**a**) and formation (**b**) processes. Black and red solid lines show experimental and successfully fitted average reaction plots, respectively. Fitting without consideration of fluctuation (cyan and broken red lines) cannot reproduce experiments.

The rate law of a first-order reaction with a rate constant k is written as $C(t)/C_0 = \exp(-kt)$, where $C(t)$ and C_0 are the concentration of the reactant at time t and 0, respectively. However, it has been known that reactions in heterogeneous systems, such as those on metal surfaces, generally exhibit environmental variations resulting in fluctuation in the rate constants, as exemplified by the site specificity of a catalytic reaction[28-32] and the geometrical variation of biological reactivity.[33-36] The rate low in these cases can be described as

$$\frac{C(t)}{C_0} = \int_0^{\infty} w(k)\exp(-kt)\mathrm{d}k$$

, where $w(k)$ is the probability density function of the rate constant. Ensemble measurements in most cases average out the microscopic details, and, consequently, $w(k)$ cannot be measured. Therefore, the observation at the microscopic scale provides a unique way to directly estimate the profile of $w(k)$ (see Fig. 2 in the main text).

A macroscopic reaction ratio can be determined from the sum of the survival probability at

the microscopic scale as

$$\frac{C(t)}{C_0}=\sum\frac{N_i(t)}{N_{0,i}}$$

, where $N_i(t)$ and $N_{0,i}$ are the numbers of the reactant molecules under the microscopic environment *i*. The survival probability is derived by integrating the probability density function of time interval, $w(\tau)$, until the reaction occurs.

$$\frac{N_i(t)}{N_{0,i}}=\int_t^\infty w_i(\tau)d\tau$$

For the first-order kinetics, i.e., $w(\tau) = k\exp(-kt)$, the survival probability follows the equation as,

$$\frac{N_i(t)}{N_{0,i}}=\exp(-k_i t)$$

Thus, the microscopic rate constant can be simply calculated as the slope of $\ln(N_i(t)/N_{0,i})$. It is mandatory for the estimation of the microscopic rate constant, k_i, that the local environment is kept constant during the measurement.[18,37-39] The present measurements achieved this requirement by avoiding the contacts between the molecular tip and substrate, which preserves the geometry and chemical compositions of the electrodes. Consequently, the linearity appeared in the reaction plots constructed from the single *I*–*t* traces (Fig. 2).

S16: Effect of surface modification on DNA hybridization.

DNA-modified substrates are often additionally covered with hydrophilic self-assembled monolayers (SAMs) in studies of DNA surface hybridization. This procedure suppresses non-specific adsorption of DNA, leading to an improvement in the hybridization efficiency. In the present study, we addressed the effect of the surface passivation on the hybridization kinetics by *I–t* measurements. The dense sample was first prepared by modification of the Au substrate with the ssDNA. The modified substrate was further immersed in 1-mM 3-mercaptopropionic acid (MPA) to construct the binary SAMs of DNA and MPA (denoted as the dense/MPA sample, Fig. S21a)[40,41]. The STM tip modified with the ssDNA, complementary to the strand in the binary SAMs, was used to obtain the *I–t* traces as in the measurements reported in the main text. We observed conductance blinking in the resulting traces, where the conductance difference between the high- and low-conductance states was calculated to be $10^{-2.8}$ G_0 (Fig. S21b). This conductance value is consistent with that observed for the diluted, dense, and ordered sample surfaces that were devoid of MPA, demonstrating the successful hybridization between the ssDNAs on the tip and on the dense/MPA sample surface.

First, we estimated the hybridization efficiency for the dense/MPA sample surface. The total lifetime of the plateaus in the *I–t* traces were normalized by the entire duration of the measurements. The efficiency was 11 ± 2%, which is smaller than the corresponding value for the dense sample (19 ± 4%; see also Fig. 5b in the main text). This tendency is not consistent with the observations made by the macroscopic ensemble measurements, where the efficiency increased by additional modification with hydrophilic molecules to the DNA-modified surface. The microscopic heterogeneity of the coexisting DNA and MPA molecules in the dense/MPA sample can explain the present result. The heterogeneity can be caused not only by the coexistence of the DNA and MPA on the surface but also by the decreased DNA surface concentration due to the exchange reaction of surface-tethered DNA and MPA during the sample preparation (see below). Fig. S21c shows the numbers of plateaus found in the consecutive *I–t* traces, obtained at the same nominal tip location (see Section S8), for the

dense/MPA and dense sample surfaces. The plots clearly show the differences in the hybridization behavior between the two samples. First, the plateaus, and thus the surface hybridization, occurred intermittently in the dense/MPA sample, whereas nearly continuous appearance of the plateaus were observed for the dense sample. Second, once the hybridization occurred, the numbers of plateaus were typically larger for the dense/MPA sample than for the dense sample. The intermittent hybridization most likely resulted in the decreased efficiency found in this study, whereas the higher occurrence probability of hybridization of the dense/MPA sample during the limited period could lead to increased efficiency in macroscopic measurements where the hybridization events occurring over the entire sample surface were simultaneously detected.

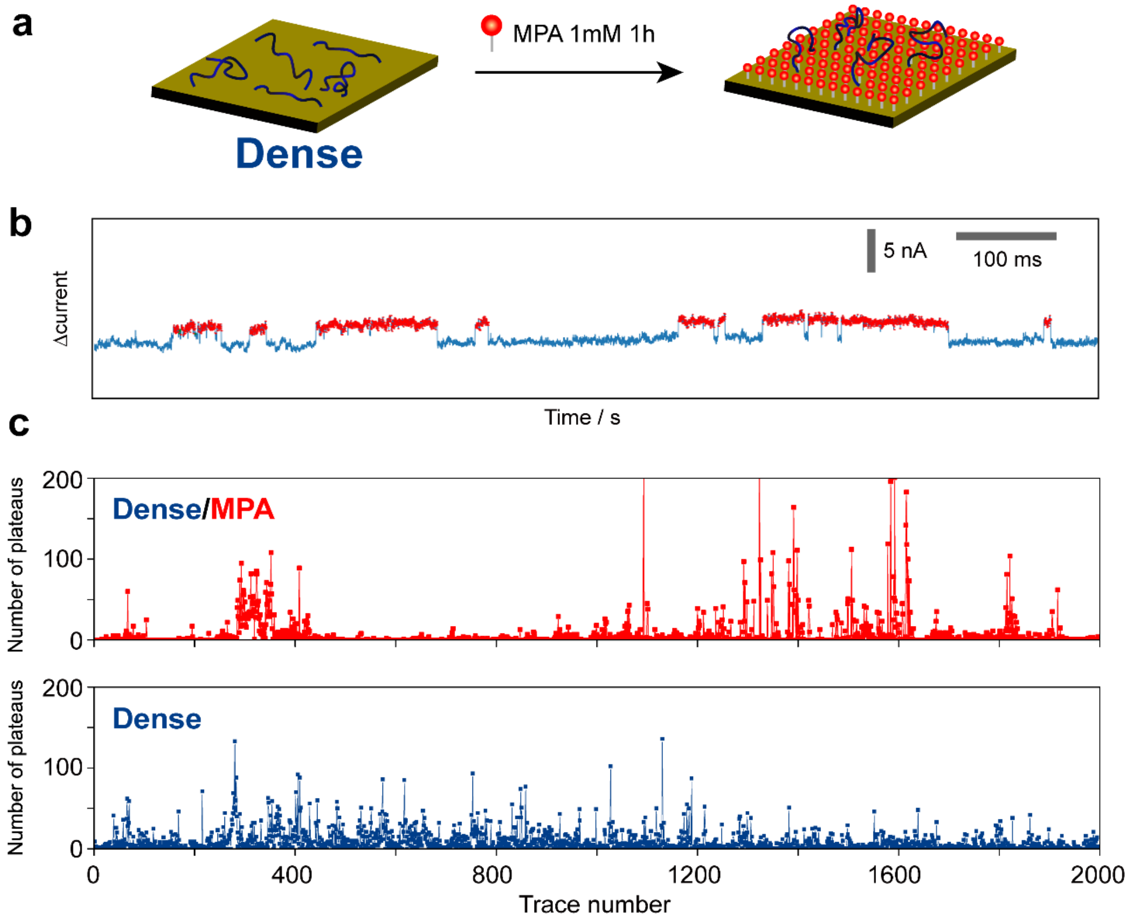

Figure S21. Hybridization behaviors on two different sample surfaces. a, Schematic illustration of preparation of dense/MPA sample. **b,** Typical result of *I*–*t* trace showing the high- and low-conducting states. Bias voltage, 20 mV; set-point current, 16 nA. **c,** Numbers

of plateaus observed during consecutive *I–t* measurements for (top) dense/MPA and (bottom) dense sample surfaces. Bias voltage of 20 mV and set-point current of 16 nA were employed in acquiring *I–t* traces.

Next, we assessed the surface hybridization on the dense/MPA sample surface from a kinetic viewpoint. In the following kinetic analyses, only *I–t* traces showing the plateaus were used to focus on the surface hybridization process. This allowed us to exclude the effect of the microscopic heterogeneity for the dense/MPA sample, which facilitated comparison with the dense sample. The kinetic model inferred from the reaction plots of the lifetime and dwell time in the *I–t* traces qualitatively agreed with those observed for the sample surfaces without MPA modification: the survival probability for lifetime decayed exponentially, whereas the survival probability for dwell time exhibited a non-linear decay (Fig. S22a). The reaction rate constants of the DNA hybridization in the DNA/MPA binary SAM were deduced from the kinetic analyses, as described in the procedures reported in the main text (Fig. S22b and c). On the basis of the kinetic models shown in Fig. 3c, the three rate constants (k_1–k_3) were calculated by the simulation-based analysis (see Fig. 4). The resulting distributions of the rate constants are shown in Fig. S22d. The average energy barriers, ΔG_1, ΔG_2, and ΔG_3, calculated for the rate constants, were found to be 64.4, 59.8, and 57.2 kJ mol^{-1}, respectively. By comparing these values with the results for the dense sample (ΔG_1 = 67.9 kJ mol^{-1}, ΔG_2 = 62.2 kJ mol^{-1}, ΔG_3 = 60.8 kJ mol^{-1}), we found that all of the energy barriers decreased for the dense/MPA sample. The energies of ΔG_1 and ΔG_2 correspond to the energy barriers for the desorption and adsorption of ssDNA strands from the surface, respectively. The decrease in ΔG_1 is attributed to the decreased adsorption energy of DNA on the surface of the hydrophilic MPA SAM compared with that on the unmodified Au surface.[42,43] Meanwhile, the DNA coverage on the dense/MPA sample was considered to be lower than that on the dense sample surface due to the exchange reaction of DNA on the surface with MPA during the MPA modification of the as-prepared dense sample.[42,43] The decreased coverage led to increased population of DNA with the lying-down conformation in the SAM, which,

in turn, accelerates surface adsorption, i.e., decreasing ΔG_2. Finally, ΔG_3 is related to the formation process of the fully hybridized dsDNA from the partially hybridized structure. The decreased interaction between DNA and the hydrophilic MPA SAM, compared with the interaction between DNA and the unmodified Au surface,[42,44-46] also explains the decrease of ΔG_3, since the weakened interaction with the surface can promote the transition from the partially to fully hybridized states. On the basis of these results, we concluded that the surface properties controlled by the deliberate functionalization affected the elementary processes of DNA surface hybridization.

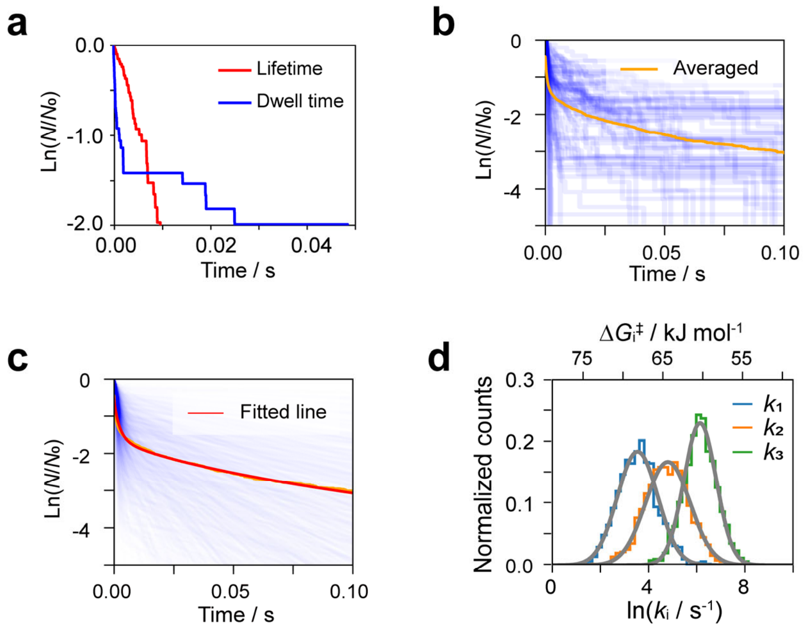

Figure S22. Kinetic analysis of surface hybridization on the dense/MPA sample surface. **a,** Reaction plots created from lifetimes (red) and dwell times (blue). **b** and **c**, Experimental and simulated reaction plots, respectively, of the dwell time. Orange and red lines show the averaged experimental and simulated plots, respectively. **d**, Distributions of k_1, k_2, and k_3 determined from the fitting procedure.

References

1 L. Herrer, A. Ismael, S. Martín, D. C. Milan, J. L. Serrano, R. J. Nichols, C. Lambert and P. Cea *Nanoscale* 2019, **11**, 15871.

2 T. Harashima, J. Yuki, T. Tsuyoshi, S. Kaneko, S. Fujii, M. Kiguchi and T. Nishino submitted for publication.

3 H. Kenzaki, N. Koga, N. Hori, R. Kanada, W. F. Li, K. Okazaki, X. Q. Yao and S. Takada *J. Chem. Theory Comput.* 2011, **7**, 1979.

4 G. S. Freeman, J. P. Lequieu, D. M. Hinckley, J. K. Whitmer and J. J. de Pablo *Phys. Rev. Lett.* 2014, **113**, 168101.

5 D. M. Hinckley, J. P. Lequieu and J. J. de Pablo *J. Chem. Phys.* 2014, **141**, 035102.

6 D. M. Hinckley, G. S. Freeman, J. K. Whitmer and J. J. de Pablo *J. Chem. Phys.* 2013, **139**, 144903.

7 E. J. Sambriski, D. C. Schwartz and J. J. de Pablo *Biophys. J.* 2009, **96**, 1675.

8 R. Krautbauer, M. Rief and H. E. Gaub *Nano Lett.* 2003, **3**, 493.

9 T. Strunz, K. Oroszlan, R. Schafer and H. J. Guntherodt *Proc. Natl. Acad. Sci. USA* 1999, **96**, 11277.

10 R. Walder, W. J. Van Patten, D. B. Ritchie, R. K. Montange, T. W. Miller, M. T. Woodside and T. T. Perkins *Nano Lett.* 2018, **18**, 6318.

11 J. D. Weeks, J. B. Lucks, Y. Kafri, C. Danilowicz, D. R. Nelson and M. Prentiss *Biophys. J.* 2005, **88**, 2752.

12 S. Kumar, J. M. Rosenberg, D. Bouzida, R. H. Swendsen and P. A. Kollman *J. Comput. Chem.* 1992, **13**, 1011.

13 Z. F. Huang, F. Chen, P. A. Bennett and N. J. Tao *J. Am. Chem. Soc.* 2007, **129**, 13225.

14 W. Haiss, C. S. Wang, I. Grace, A. S. Batsanov, D. J. Schiffrin, S. J. Higgins, M. R. Bryce, C. J. Lambert and R. J. Nichols *Nat. Mater.* 2006, **5**, 995.

15 B. P. English, W. Min, A. M. van Oijen, K. T. Lee, G. Luo, H. Sun, B. J. Cherayil, S. C. Kou and X. S. Xie *Nat. Chem. Biol.* 2006, **2**, 87.

16 M. Lippitz, F. Kulzer and M. Orrit *Chemphyschem* 2005, **6**, 770.

17 H. P. Lu, L. Xun and X. S. Xie *Science* 1998, **282**, 1877.

18 Y. Li, H. Wang, Z. Wang, Y. Qiao, J. Ulstrup, H.-Y. Chen, G. Zhou and N. Tao *Proc. Natl. Acad. Sci. USA* 2019, **116**, 3407.

19 A. Hamelin *J. Electroanal. Chem.* 1996, **407**, 1.

20 A. W. Peterson *Nucleic Acids Res.* 2001, **29**, 5163.

21 I. Y. Wong and N. A. Melosh *Nano Lett.* 2009, **9**, 3521.

22 D. Y. Petrovykh, H. Kimura-Suda, L. J. Whitman and M. J. Tarlov *J. Am. Chem. Soc.* 2003, **125**, 5219.

23 A. N. Rao and D. W. Grainger *Biomater. Sci.* 2014, **2**, 436.

24 X. Jin, Z. Ma, J. Talbot and N.-H. L. Wang *Langmuir* 1999, **15**, 3321.

25 C.-Y. Lee, P. Gong, G. M. Harbers, D. W. Grainger, D. G. Castner and L. J. Gamble *Anal. Chem.* 2006, **78**, 3316.

26 D. Y. Petrovykh, V. Pérez-Dieste, A. Opdahl, H. Kimura-Suda, J. M. Sullivan, M. J. Tarlov, F. J. Himpsel and L. J. Whitman *J. Am. Chem. Soc.* 2006, **128**, 2.

27 S. Cocco, R. Monasson and J. F. Marko *Phys. Rev. E* 2002, **65**, 041907.

28 A. Bruix, J. T. Margraf, M. Andersen and K. Reuter *Nat. Catal.* 2019, **2**, 659.

29 I. L. C. Buurmans and B. M. Weckhuysen *Nat. Chem.* 2012, **4**, 873.

30 M. J. S. Farias, W. Cheuquepan, G. A. Camara and J. M. Feliu *Acs Catal.* 2016, **6**, 2997.

31 K. Tedsree, T. Li, S. Jones, C. W. A. Chan, K. M. K. Yu, P. A. J. Bagot, E. A. Marquis, G. D. W. Smith and S. C. E. Tsang *Nat. Nanotechnol.* 2011, **6**, 302.

32 T. Zambelli, J. Wintterlin, J. Trost and G. Ertl *Science* 1996, **273**, 1688.

33 Z. J. Deng, M. Liang, M. Monteiro, I. Toth and R. F. Minchin *Nat. Nanotechnol.* 2010, **6**, 39.

34 W. Shang, J. H. Nuffer, J. S. Dordick and R. W. Siegel *Nano Lett.* 2007, **7**, 1991.

35 M. Gebala and W. Schuhmann *Chemphyschem* 2010, **11**, 2887.

36 U. Rant, K. Arinaga, S. Scherer, E. Pringsheim, S. Fujita, N. Yokoyama, M. Tornow and G. Abstreiter *Proc. Natl. Acad. Sci. USA* 2007, **104**, 17364.

37 E. Kazuma, J. Jung, H. Ueba, M. Trenary and Y. Kim *J. Am. Chem. Soc.* 2017, **139**, 3115.

38 E. Kazuma, J. Jung, H. Ueba, M. Trenary and Y. Kim *Science* 2018, **360**, 521.

39 J. Guan, C. Jia, Y. Li, Z. Liu, J. Wang, Z. Yang, C. Gu, D. Su, K. N. Houk, D. Zhang and X. Guo *Sci. Adv.* 2018, **4**, eaar2177.

40 V. Dharuman and J. H. Hahn *Sens. Actuators B Chem.* 2007, **127**, 536.

41 M. Satjapipat, R. Sanedrin and F. Zhou *Langmuir* 2001, **17**, 7637.

42 T. M. Herne and M. J. Tarlov *J. Am. Chem. Soc.* 1997, **119**, 8916.

43 R. Levicky, T. M. Herne, M. J. Tarlov and S. K. Satija *J. Am. Chem. Soc.* 1998, **120**, 9787.

44 J. Liu *Phys. Chem. Chem. Phys.* 2012, **14**, 10485.

45 J. Wang, X. Guo, R. Liu, J. Guo, Y. Zhang, W. Zhang and S. Sang *Nanotechnology* 2020, **31**, 015501.

46 M. Chen, T. T. Nguyen, N. Varongchayakul, C. Grazon, M. Chern, R. C. Baer, S. Lecommandoux, C. M. Klapperich, J. E. Galagan, A. M. Dennis and M. W. Grinstaff *Adv. Healthc. Mater.* 2020, **9**, 2000403.
